# Essential Role of LapD in the Absence of Cardiolipins

**DOI:** 10.3390/ijms27031445

**Published:** 2026-01-31

**Authors:** Satish Raina, Akshay Maniyeri, Aravind Ayyolath, Gracjana Klein

**Affiliations:** Laboratory of Bacterial Genetics, Gdansk University of Technology, 80-233 Gdansk, Poland

**Keywords:** lipopolysaccharide (LPS), phospholipids, LapD (YhcB), cardiolipin, phosphatidylglycerol, thioesterase, LapB, PgsA, acetyl-CoA carboxylase (ACC)

## Abstract

To maintain the integrity of the outer membrane of Gram-negative bacteria, such as *Escherichia coli*, the levels of two essential components, phospholipids (PL) and lipopolysaccharide (LPS), are tightly regulated, although the underlying molecular mechanisms are unclear. *E*. *coli* synthesizes three main PLs, including essential phosphatidylethanolamine and phosphatidylglycerol and nonessential cardiolipin (CL). We showed that CL synthesis is conditionally essential in Δ*lapD* bacteria. Using this synthetic lethal phenotype, we isolated suppressors that rescued growth at elevated temperatures. We showed that loss-of-function mutations in *cdsA* encoding CDP-diglyceride synthetase, and *pgsA*, which encodes phosphatidylglycerophosphate synthase, bypass this lethality. Such mutations reduce the relative abundance of acidic phospholipids, which are otherwise elevated in Δ(*lapD clsA*) bacteria, and increase the amounts of *cis*-vaccenic acid without altering amounts of LpxC mediating the first committed step in LPS biosynthesis. Interestingly, overexpression of genes, including *accC* and *glnB*, whose products can inhibit fatty acid/PL synthesis, overcame the lethality of Δ(*lapD clsA*) bacteria. We demonstrated that PgsA co-purifies with LapB, which regulates LpxC stability and acts as a hub for proteins involved in PL and LPS biosynthesis, including LapD. Overall, our results reveal that LapD is positioned at the regulatory nexus between LPS assembly and fatty acid/PL synthesis.

## 1. Introduction

The cell envelope of Gram-negative bacteria, including *Escherichia coli*, contains two distinct membranes, an inner (IM) and an outer (OM) membrane, separated by the periplasm, which includes a thin layer of peptidoglycan. The defining feature of the OM of Gram-negative bacteria is its asymmetry due to the presence of phospholipids (PLs) in the inner leaflet and the location of lipopolysaccharide (LPS) in the outer leaflet [1]. LPS constitutes the major component of the OM, and its essentiality is due to its role in providing a permeability barrier and structural integrity of the OM [2]. Similarly, PLs are integral in regulating membrane integrity and its fluidity [3]. Both of these components of OM are essential for bacterial viability, and their amounts are tightly regulated, as these two essential components use the same *β*-hydroxymyristoyl-ACP as a common metabolic precursor [4,5]. Hence, any imbalance between LPS and PLs leads to a loss of bacterial viability. Thus, bacteria, such as *E*. *coli*, hold a constant ratio of 0.15:1.0 for these two essential components, respectively [6]. However, the mechanisms by which bacteria maintain homeostatic control are poorly understood. One of the most studied regulatory controls that has received recent attention is the regulated turnover of LpxC, which catalyzes the first committed step in LPS biosynthesis [7]. This involves the positive regulation of LpxC proteolysis by the FtsH-LapB complex and its negative control by LapC [5,8,9]. PLs are derived from fatty acids (FA). Bacteria, such as *E. coli*, have devised controls for the biosynthesis of new fatty acids and the modification of the structure of existing fatty acids [3]. Similarly, LPS can undergo structural non-stoichiometric modifications in its lipid A and core parts, which impart resistance to cationic antibiotics [10,11,12]. Together, these processes allow bacteria to adjust LPS amounts according to their demand and control membrane viscosity to match environmental requirements. Another player, LapD, has recently been implicated in the regulation of LPS and fatty acid balance, although the precise mechanism of its involvement remains unknown [13,14].

**Figure 1:**
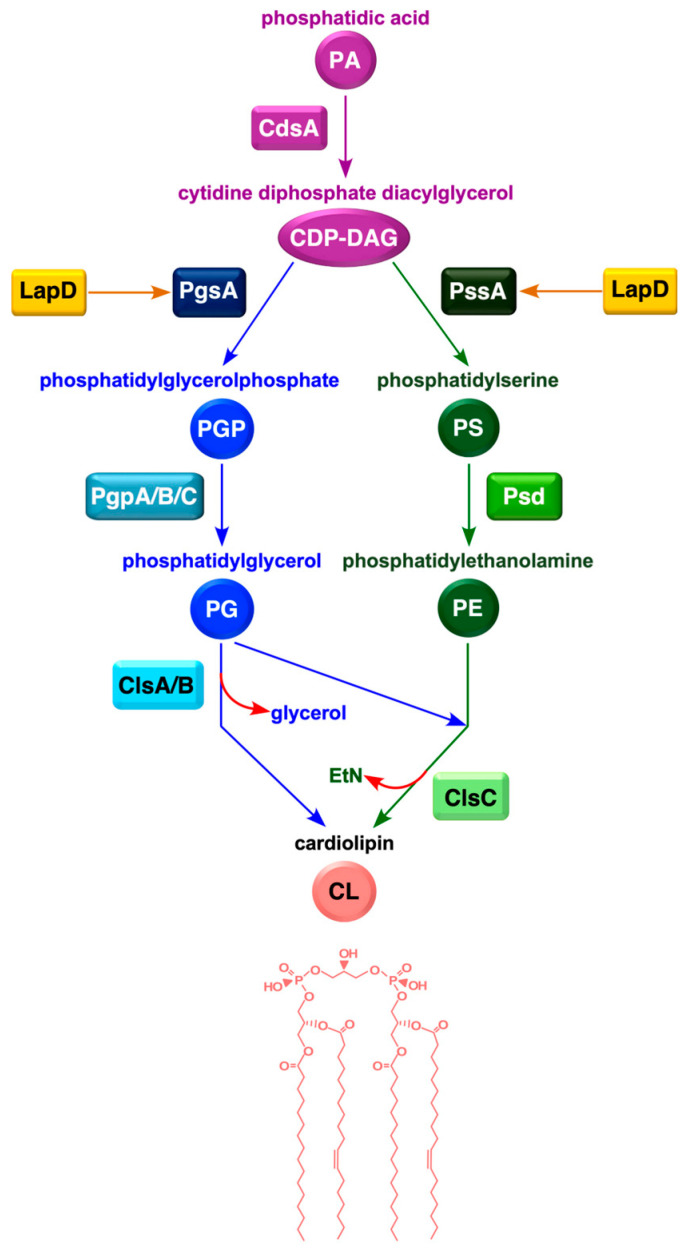
Schematic drawing of the phospholipid biosynthetic pathway in *E*. *coli*. The key intermediate in PL synthesis is cytosine diphosphate-diacylglycerol (CDP-DAG), which is synthesized by CdsA from phosphatidic acid. The major PL and PE are synthesized in two steps, as shown. PG molecules are also synthesized from CDP-DAG using PgsA to form phosphatidylglycerolphosphate, which is then dephosphorylated by Pgp enzymes. CL molecules are synthesized by the condensation of either two PG molecules or a single PE and PG molecule, as indicated. Co-purification of LapD with PgsA and PssA are indicated by arrows.

In *E. coli* LPS biosynthesis, a tetraacylated lipid A precursor is first synthesized via a pathway that requires six essential enzymes [2,15]. Two Kdo residues are added to lipid IV_A_ by the WaaA enzyme, followed by the addition of secondary lauroyl and myristoyl chains, resulting in the synthesis of hexaacylated lipid A. Additional sugars are added to this key intermediate, leading to the LPS core formation. In smooth bacteria, a distal *O*-antigen is further attached after flipping LPS across the IM on the periplasmic side [15]. The synthesis of hexaacylated LPS is crucial because LPS that is either tetraacylated or pentaacylated is poorly translocated to the OM [16,17]. Furthermore, the absence of myristoyl acyltransferase LpxM causes synthetic lethality when either LPS or PL synthesis is compromised [13,14,18,19]. The fatty acid biosynthesis pathway in *E. coli* is of the type FASII system that comprises four steps using both acetyl coenzyme A (acetyl-CoA) and malonyl-ACP as starter units and malonyl-ACP as the extender unit [3]. In the initiation of FA biosynthesis, acetyl-CoA carboxylase (ACC) catalyzes the first committed step in fatty acid biosynthesis: the conversion of acetyl-CoA to malonyl-CoA [20,21]. Each turn of the FA biosynthesis cycle adds two carbon atoms to the growing acyl chain. The long-chain acyl-ACP thioesters generated during FA synthesis serve as acyl donors for PLs synthesis [22]. Phosphatidic acid (PA) serves as a universal precursor for membrane PL formation [23]. In *E*. *coli*, PA is generated by the acylation of *sn*-glycerol-3-phosphate (G3P) to 1-acyl-G3P, followed by a second acylation, requiring PlsB and PlsC acyltransferases, respectively [23,24]. PA is then converted to the central precursor of all phospholipids, cytidine diphosphate-diacylglycerol (CDP-DAG), by CDP-DAG synthase encoded by the *cdsA* gene (Figure 1). The primary PLs in *E. coli* are phosphatidylethanolamine (PE), phosphatidylglycerol (PG), and cardiolipin (CL). PE and PG are essential for bacterial viability, whereas CL is dispensable. CDP-DAG is positioned at a branch point in PL biosynthesis, where it reacts with *sn*-glycerol 3-phosphate to form PG or with L-serine to synthesize phosphatidylserine (Figure 1) [25]. CL is produced via the condensation of two PG molecules or a single PE and PG molecule [22]. The balance of zwitterionic (PE) and acidic phospholipids (PG and CL) in *E. coli* stems from the continued synthesis of PG by the PG phosphate synthase–phosphatase (PgsA–PgpA/B) system, which is coupled with the tight regulation of PE synthesis through the PssA–Psd pathway [3] (Figure 1).

*E. coli* has three CL synthases, with ClsA being the major contributor for producing CL. Although nonessential for bacterial viability, CL plays a crucial role in maintaining structural integrity, cell size, and regulating the activity of various proteins, such as the polar localization of ProP, assembly of LacY, and protein transport through the Sec apparatus [26,27,28]. More importantly, it was shown that CL presence is essential for the viability of strains lacking either WaaA Kdo transferase or LpxM myristoyl acyltransferase [19]. As suppressors that overcome the synthetic lethality of Δ(*clsA lpxM*) mapped to MsbA LPS flippase, it was proposed that CL participates in LPS translocation [18,19].

Biochemical characterization of LapD revealed that it co-purifies with enzymes involved in PL, FA, and LPS biosynthesis. Although LapD is non-essential, its absence confers synthetic lethality when lipid A is underacylated or when LPS is composed only of Kdo_2_-lipid A [13,14]. Δ(*lapD lpxM*) bacteria were found to contain elevated amounts of acidic phospholipids and significant alterations in FA composition, supporting a role of LapD in regulation of FA composition. Consistent with this hypothesis, suppressor mutations that overcome the lethal phenotype of Δ(*lapD lpxM*) were found to map to genes encoding various subunits of the ACC complex, which restored PL amounts. However, the absence of LapD also causes retention of LPS in the IM, and suppressors that relieve the conditional lethal phenotype of Δ(*lapD waaC*) bacteria map to either genes involved in LPS assembly in the OM (*lptD*) or in the *nlpI* gene, whose product regulates proteolysis of peptidoglycan hydrolase [14]. Thus, LapD is an enigmatic protein, and significant gaps remain in our understanding of the pleiotropic phenotypes associated with its absence.

**Figure 2:**
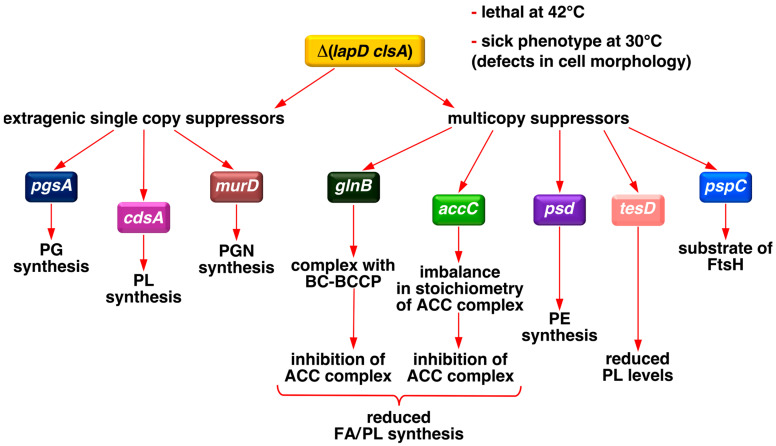
Schematic drawing of the approaches used to address the molecular basis of the lethality of Δ(*lapD clsA*) bacteria. Extragenic single-copy suppressors that restore growth at elevated temperatures were mapped to either PL biosynthetic (*pgsA* and *cdsA*) genes or those involved in peptidoglycan (PGN) formation (*murD*). Identification of multicopy suppressors revealed that either the inhibition of FA/PL biosynthesis (*glnB* and *accC*), reduction in PL amounts (*tesD*), or titration of the FtsH protease (*pspC*) can overcome this lethality. GlnB forms a complex with biotin carboxylase/biotin carboxyl carrier protein (BC-BCCP), which inhibits the acetyl-CoA carboxylase (ACC) enzyme.

In this study, we showed that Δ(*lapD clsA*) bacteria exhibited conditional lethality. Thus, we used this synthetic lethal phenotype of Δ(*lapD clsA*) to isolate single-copy chromosomal and multicopy suppressors that overcame this lethality and addressed their cell morphology defects and PL/fatty acid composition (Figure 2). Most single-copy extragenic suppressors mapped to genes involved in PL biosynthesis. These include single amino acid exchanges in the *pgsA* gene, whose product catalyzes the condensation of CDP-DAG with G3P to form the intermediate PG phosphate (PGP), which is then dephosphorylated by Pgp phosphatases to produce PG (Figure 1). Another set of suppressors carried a mutation mapping to the essential *cdsA* gene, which encodes CDP-DAG synthetase that catalyzes the synthesis of CDP-DAG [29]. Mutations in *pgsA* and *cdsA* genes reduced PL levels, which are otherwise elevated in Δ(*lapD clsA*) bacteria, and restored the fatty acid composition. Identification and characterization of multicopy suppressors revealed that overexpression of genes (*accC* and *glnB*), whose products inhibit fatty acid biosynthesis at the level of the ACC enzyme, or the *tesD* gene encoding a putative thioesterase, which causes a decrease in the overall PL content, overcomes the lethality of Δ(*lapD clsA*) bacteria. We further showed that PgsA co-purifies with LapB/D complex and that Δ(*lapD clsA*) bacteria exhibit gross alterations in cellular morphology, which are suppressed by loss-of-function mutations in the *pgsA* gene. These genetic and biochemical studies revealed that the lethality of Δ(*lapD clsA*) is due to the toxic accumulation of PL moieties and that LapD acts as a nodal point in the homeostatic control of FA and LPS biosynthesis.

## 2. Results

### 2.1. Suppressor-Free Δ(lapD clsA) Bacteria Exhibit Conditional Synthetic Lethality

The function of LapD is not well understood but has been implicated in cell envelope-related functions, including a role in maintaining a balance between LPS and PLs. Thus, we undertook several experiments to address the role of LapD when PL biosynthesis is impaired. One of the non-essential PL components is CL, but its cellular requirement is not well understood. The major enzyme required for cardiolipin formation under exponential growth conditions is ClsA [30,31]. Individually, *lapD* and *clsA* genes are dispensable for bacterial viability under laboratory growth conditions. However, as shown earlier, the *clsA* gene becomes essential when LPS is underacylated and exhibits a sick phenotype in the absence of LapD [13]. We reconstructed Δ(*lapD clsA*) strains in the W3110 background, which has been extensively used for LPS and PL analysis, because some other background strains, such as BW25113, carry a non-functional *fabR* gene [32]. Thus, reciprocal bacteriophage P1-mediated transductions were carried out in the wild type and its isogenic derivatives lacking either *lapD* or *clsA* genes with the vector alone or with a covering plasmid. Parallel transductants were plated on LA plates at 30, 37, and 42 °C. The results from these experiments revealed that Δ*lapD* and Δ*clsA* can be introduced into the wild type at the same frequency at the three temperatures. However, Δ(*lapD clsA*) or reciprocal Δ(*clsA lapD*) viable transductants could be obtained at 30 °C with or without covering the plasmid, although the transductants were obtained at a lower frequency (Table 1). These transductants exhibited a small colony morphology. At 42 °C, no viable Δ(*lapD clsA*) and Δ(*clsA lapD*) transductants could be obtained without covering the plasmid (Table 1). At 37 °C, transductants could be obtained but at a highly reduced frequency. These results allow us to conclude that the Δ(*lapD clsA*) combination is synthetically lethal at 42 °C and, although viable at 30 °C, exhibits a sick phenotype.

### 2.2. Suppressors That Allow Δ(lapD clsA) Bacterial Growth at Elevated Temperatures Map to Phospholipid Biosynthetic Genes pgsA, cdsA, and Those Involved in Peptidoglycan Biosynthesis

**Figure 3:**
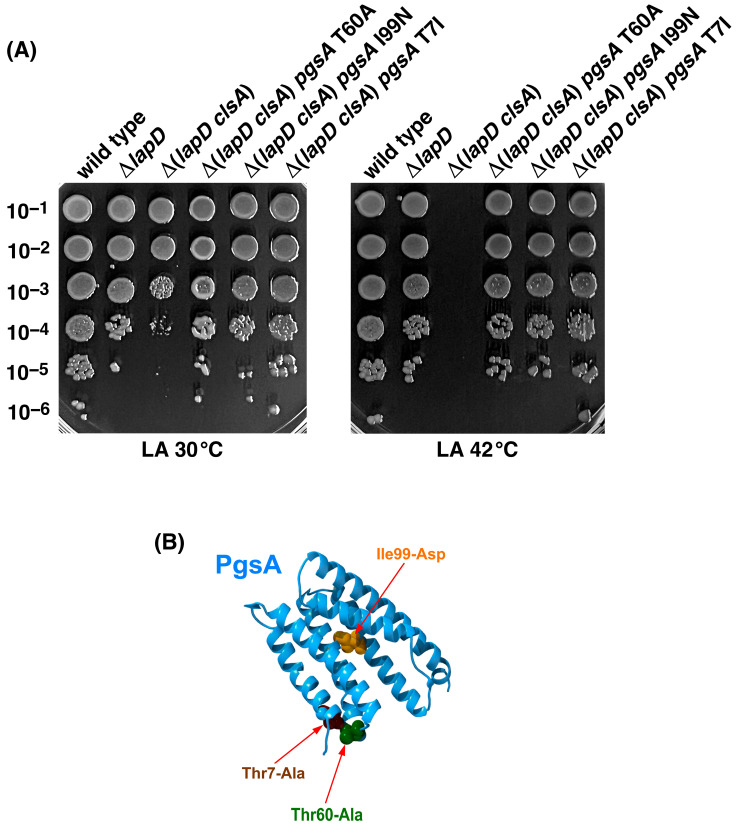
Mutations mapping to the *pgsA* gene encoding phosphatidylglycerophosphate synthase overcome the synthetic lethality of Δ(*lapD clsA*) bacteria. (**A**) Exponentially grown cultures of the wild type, Δ*lapD*, and Δ(*lapD clsA*) derivatives with and without suppressor mutations mapping to the *pgsA* gene grown at 30 °C. The cultures were adjusted to an OD_595_ of 0.1 and serially diluted. Bacterial growth was estimated by spot dilution on LA media at 30 and 42 °C. The relevant genotypes and incubation temperatures are indicated. (**B**) Positions of various single amino acid substitutions in the structure of PgsA AlphaFold AF-P0ABF8-F1 are shown.

To address the molecular basis of the conditional synthetic lethality of Δ(*lapD clsA*) bacteria, extragenic suppressors that allow growth at 42 °C were sought by plating cultures at elevated temperatures. Temperature-resistant (Ts^+^) survivors were obtained at a frequency of approximately 10^−8^. Ts^+^ independently obtained suppressors were marked with the Tn*10* transposon, as described previously [14]. Linked Ts^+^ mutations were verified by retransducing the suppressor mutation into the parental Δ(*lapD clsA*) strain SR25209. Fourteen independent suppressors with transposon linked >90% were retained after verification. To map the suppressor mutations, Tn*10* insertions were recombined onto cosmids. The identity of the suppressor mutation was determined by sequencing the PCR products of the complementing cosmids. These mutations could be grouped into three complementation groups, with 10 out of 14 linked to the *uvrC* gene. The second complementation group of three strains with the suppressor mutation was linked to the *skp* gene. The third complementation group had Tn*10* mapping to the *mraZ* gene, located next to the gene cluster carrying genes whose products are involved in cell division and peptidoglycan biosynthesis. DNA sequencing of the region next to *uvrC*, *skp*, and *mraZ* genes revealed single amino acid exchanges in the essential *pgsA*, *cdsA*, and *murD* genes, respectively. These results further showed that five independently isolated suppressors had the suppressor mutation in the *pgsA* gene encoding phosphatidylglycerophosphate synthase, causing alteration of aa T60. Four out of five had a single amino acid exchange of T60A (ACC to GCC) in the *pgsA* gene (Table 2). Another suppressor-carrying strain had a single amino acid alteration, T60P, in the coding region of the *pgsA* gene. Significantly, the T60A mutation results in the alteration of aa 60, which is the same residue with a substitution to be the first reported and very well characterized *pgsA3* mutation [33,34,35]. However, the original *pgsA* has an exchange of T60P, as opposed to our T60A substitution, which was found in four out of five independently obtained Ts^+^ suppressors. Significantly, it is encouraging that we also obtained one suppressor mutation that is identical to the original *pgsA3* mutation. The *pgsA3* mutation in the *pgsA* gene lacks the potential to synthesize phosphatidylglycerolphosphate, a precursor of PG [36]. The original *pgsA3* was identified as a Ts^+^ suppressor mutation of the (*pssA-1 clsA*) strain [36]. Similarly to the *pgsA3* mutation, *pgsA* T60A was found to be recessive, as it could be complemented by a cosmid clone carrying the *pgsA* gene. Further DNA sequence analysis revealed that three strains carried a single amino acid substitution of T7A (ACG to GCG) in the coding region of the *pgsA* gene, and the two remaining strains carried a single amino acid substitution of I99N at aa position 99 due to ATC to AAC nucleotide alteration (Table 2). All three amino acid residues, T60, T7, and I99, are highly conserved in PgsA orthologs, and substitution of T60 has been shown to cause a loss of enzymatic activity (Figure 3).

The complementation group linked to the *skp* gene, comprising three Ts^+^ suppressor-bearing strains, had a single amino acid substitution at aa position 249 due to GAC to GCC alteration in the coding region of the *cdsA* gene, resulting in the D249G substitution. The *cdsA* gene encodes CDP-diglyceride synthetase, which catalyzes the synthesis of CDP-diacylglycerol (CDP-DAG) using CTP and phosphatidic acid (PA) as substrates [29,37]. As CDP-DAG serves as a precursor of all PLs in *E*. *coli*, we expect that the suppressor mutation could act by reducing the overall PL amounts. DNA sequencing analysis of the adjoining regions of the Tn*10* mutation revealed that the last Ts suppressor of Δ(*lapD clsA*) carries a suppressor mutation in the essential *murD* gene due to the exchange of M407V (ATG to GTG). The *murD* gene encodes UDP-*N*-acetylmuramoyl-L-alanine:D-glutamate ligase, which catalyzes the addition of the second amino acid to the peptide moiety of the monomer unit of peptidoglycan [38]. The amino acid residue 407 is located in the conserved C-terminal domain, which could be important for the stability and specificity of the MurD enzyme [38].

### 2.3. Evaluation of Growth Properties of Suppressors That Overcome Synthetic Lethality of Δ(lapD clsA) Bacteria at Elevated Temperatures

**Figure 4:**
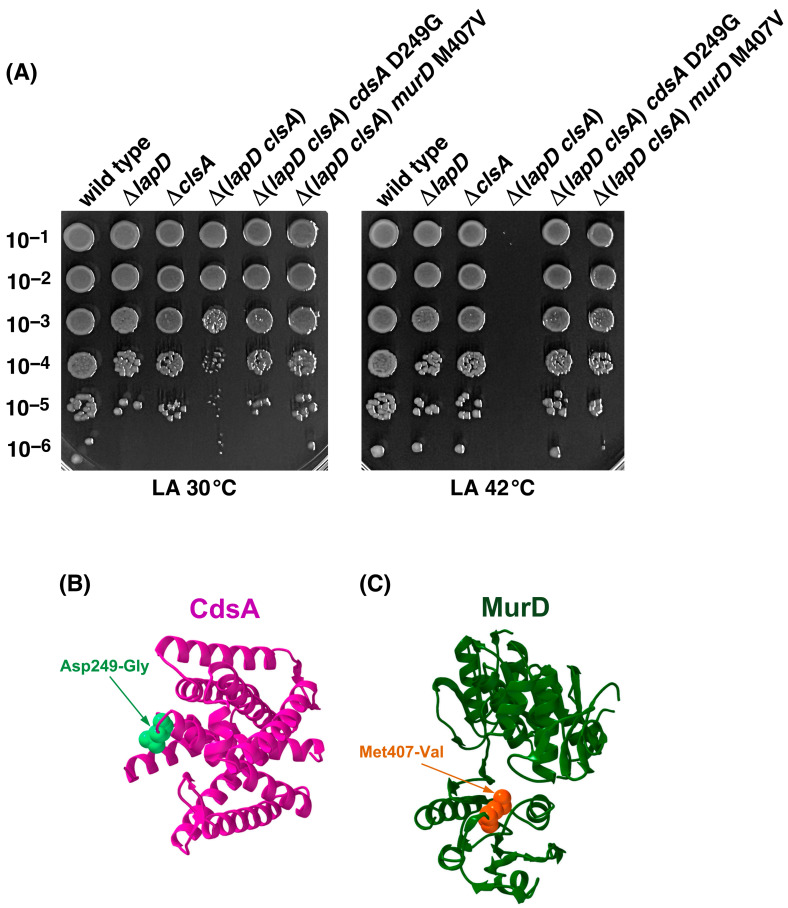
Mutations mapping to *cdsA* and *murD* genes overcome the synthetic lethality of Δ(*lapD clsA*) bacteria at 42 °C. (**A**) Growth of isogenic cultures of the wild type, Δ*lapD*, Δ*clsA*, and Δ(*lapD clsA*) derivatives with and without suppressor mutations mapping to various *cdsA* and *murD* genes was quantified by spot dilution on LA media at 30 and 42 °C. The relevant genotypes and incubation temperatures are indicated. (**B**) Positions of various single amino acid substitutions in the structure of CdsA AlphaFold AF-P0ABG1-F1, (**C**) MurD PDB 1E0D are shown.

As the majority of single-copy suppressor mutations mapped to the *pgsA* gene that overcame the synthetic lethality of Δ(*lapD clsA*) bacteria, we estimated their growth properties using a spot-dilution assay at 30 and 42 °C. The wild type, Δ*lapD*, and isogenic strains with suppressor mutations in the *pgsA* gene showed normal growth at 30 and 42 °C. However, Δ(*lapD clsA*) bacteria exhibited a 10-fold reduction in colony-forming ability, even at 30 °C, and lethality at 42 °C (Figure 3). Thus, the Δ(*lapD clsA*) combination conferred a sick phenotype at 30 °C, with a severe reduction in the colony size. Furthermore, Δ(*lapD clsA*) bacteria are unable to grow at elevated temperatures and is synthetically lethal at 42 °C. In parallel, we quantified the growth properties of Δ(*lapD clsA*) derivatives with suppressor mutations mapping to *cdsA* and *murD* genes at 30 and 42 °C. The spot-dilution assay again revealed the sick phenotype of Δ(*lapD clsA*) bacteria at 30 °C and the lethal phenotype at 42 °C. Importantly, suppressor mutations in either *cdsA* or *murD* genes restored nearly wild-type-like growth at 42 °C, validating their isolation (Figure 4).

### 2.4. Restoration of Phosphatidylglycerol Levels in Δ(lapD clsA) Bacteria by Suppressor Mutations in PgsA

**Figure 5:**
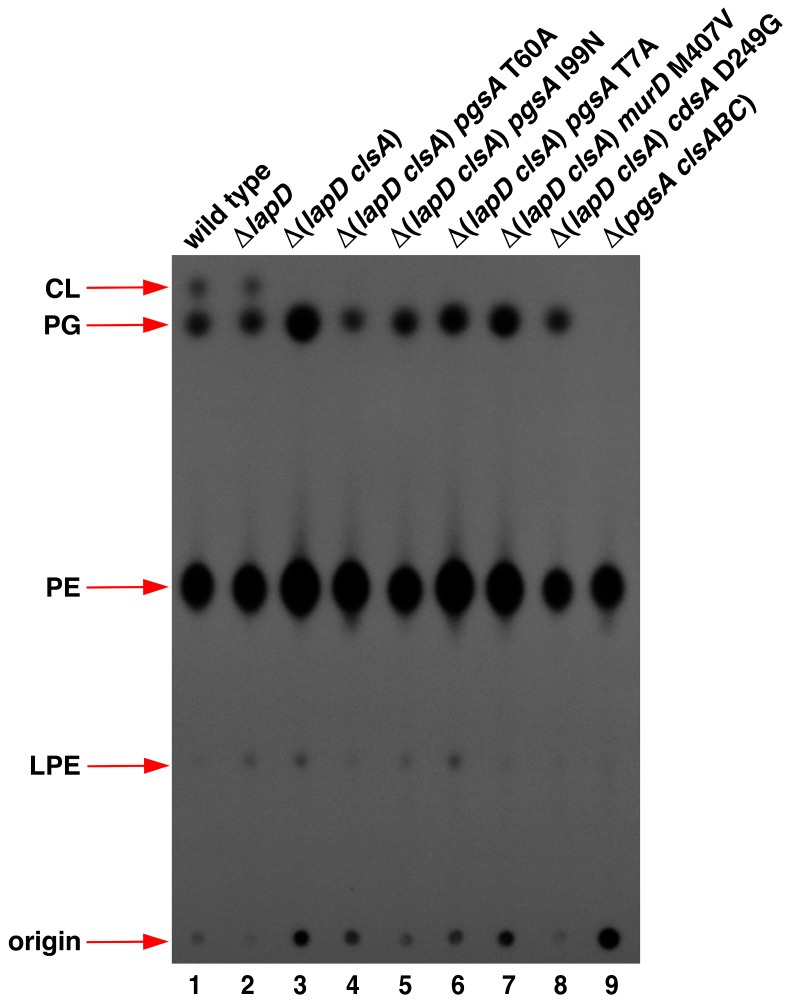
Δ(*lapD clsA*) bacteria have excess PG and PE species of phospholipids and suppressor mutations in *pgsA* and *cdsA* genes, whose products are involved in PL biosynthesis, which overcome their synthetic lethality by decreasing PG and/or PE synthesis. Thin-layer chromatography of ^32^P-labeled PLs extracted from isogenic strains derived from Δ(*lapD clsA*) bacteria carrying suppressor mutations in *pgsA*, *murD*, and *cdsA* genes. In parallel, ^32^P-labeled PLs were extracted from the parental wild-type and Δ*lapD* bacteria, with samples from the RU857 strain serving as a marker for the migration of PL species. A representative image from three biological replicates is shown. The arrows indicate the positions of the major PL species.

To address the molecular basis of the synthetic lethality of Δ(*lapD clsA*) bacteria, the PL composition of panels of strains with and without suppressor mutations in *pgsA*, *cdsA*, and *murD* genes was analyzed using thin-layer chromatography (TLC). PL were extracted as a control from the strain RU857, which cannot synthesize cardiolipin (CL) and phosphatidylglycerol (PG) species and serves as an internal marker for various PL species. The cultures were labeled with inorganic phosphate, and PL was extracted as described previously [14]. Equivalent amounts of total radiolabeled PL were applied after adjusting for the total protein content. PL species were visualized using phosphorimaging analysis and quantified by densitometry. Densitometric quantification of different PL species revealed that the wild-type strain contained approximately 74.30% ± 3.9 PE, followed by 19.15% ± 0.8 PG and 6.54% ± 0.5 CL (Figure 5, lane 1). These values are consistent with those of the three known PL species in *E. coli* [39]. No major differences in the composition of PL species were observed between the extracts obtained from wild-type and Δ*lapD* bacteria, except for the minor accumulation of lysophosphatidylethanolamine (LPE) in Δ*lapD* and in some of its derivatives (Figure 5, lane 2). However, most significantly, Δ(*lapD clsA*) contained approximately a 4.2-fold increase in PG and a 1.5-fold increase in PE compared to the wild type (Figure 5, lane 3). Interestingly, the isogenic strain Δ(*lapD clsA*) with the *pgsA* T60A suppressor mutation repressed this vast accumulation of PG, reducing it by 5.9-fold compared to the wild-type level (Figure 5, lane 4). Other suppressor mutations, *pgsA* I99N and *pgsA* T7A, also reduced the elevated accumulation of PG amounts (Figure 5, lanes 5 and 6), although not as dramatically as observed with the *pgsA* T60A suppressor mutation. The suppressor mutation *pgsA* I99N reduced PG levels by nearly 1.8-fold compared to those in the parental Δ(*lapD clsA*) strain. The suppressor mutation *cdsA* D249G reduced elevated PE levels by more than 15% compared to that in the wild type (Figure 5, lane 8). However, no major differences were observed with the suppressor mutation in the *murD* gene, which is not expected to directly regulate PL amounts and would likely only regulate PGN. Thus, we can conclude that suppressor mutations in either *pgsA* or *cdsA* genes relieve the lethality of Δ(*lapD clsA*) by reducing the toxic accumulation of elevated PG and PE levels.

### 2.5. Δ(lapD clsA) Bacteria Exhibit Gross Alterations in Fatty Acid Composition, Which Are Suppressed by Mutations in PgsA

It is well established that fatty acid and PL synthesis are intricately linked. In the above sections, we showed that Δ(*lapD clsA*) bacteria have significantly altered amounts of PL species, particularly with hyperelevated amounts of PG. Thus, we further analyzed the fatty acid composition of Δ(*lapD clsA*) bacteria and their isogenic suppressors that overcome their lethal phenotype. Cultures were grown in LB medium, and free fatty acids (FFA) were extracted from an isogenic panel of strains grown at 28 and 37 °C and analyzed using gas chromatography (GC).

GC quantification of FFA extracted from cultures grown at 28 °C revealed that most significantly Δ(*lapD clsA*) had approximately 20% less unsaturated fatty acid (UFA) species 18:1 *cis*-vaccenic acid than the wild type (Table 3). The most robust suppressor mutation in Δ(*lapD clsA*) *pgsA* T60A reversed this composition, leading to an increase in more than 23% in 18:1 *cis*-vaccenic acid content compared to that in the wild type. Interestingly, the suppressor mutations *pgsA* T7A and *cdsA* D249G restored 18:1 levels to nearly wild-type levels (Table 3). Furthermore, the Δ(*lapD clsA*) strain accumulated 3-fold more amounts of saturated fatty acid 18:0 as compared to the parental strain, which was partly reversed by the *pgsA* T60A mutation. Significantly, Δ(*lapD clsA*) *pgsA* T60A bacteria accumulated only 58% myristic acid (14:0). Another major difference is an approximately 30% decrease compared to the wild type in the amount of 16:1 palmitoleic acid UFA when *pgsA* T60A is present in Δ(*lapD clsA*) bacteria at 28 °C.

Analysis of GC data from FFA extracted from bacteria grown at 37 °C further reinforces the results from 28 °C that the severe growth defects of Δ(*lapD clsA*) bacteria are due to gross alterations in the fatty acid composition. The amount of UFA *cis*-vaccenic acid 18:1 species was even more reduced, being approximately 64% of that in the wild type (Table 3). This was reversed by suppressor mutations in *pgsA* and *cdsA* genes. The suppressor mutation *pgsA* T60A caused more than a 32% increase in the 18:1 UFA content compared to the wild type. Notably, Δ(*lapD clsA*) bacteria exhibited an increased presence of 12:0 and 14:0 saturated fatty acids, which were repressed by some of the suppressor mutations, particularly for myristic acid (14:0). Taken together, the results from the GC experiments allow us to conclude that the absence of CL in Δ*lapD* bacteria results in large-scale changes in the biosynthesis of fatty acids, some of which are reversed by suppressor mutations in *pgsA* and *cdsA* genes. Thus, these results can explain the molecular basis of the synthetic lethality of the Δ(*lapD clsA*) combination and provide a rationale for the isolation of suppressor mutations in the PL biosynthetic pathway. These results are consistent with the known bacterial requirement to maintain a balance between saturated and unsaturated fatty acids, which is critical for membrane fluidity, and in this process, LapD and ClsA functions are critical.

### 2.6. Δ(lapD clsA) Bacteria Have Severe Defects in Cell Morphology, Which Are Suppressed by Mutations in PgsA

**Figure 6:**
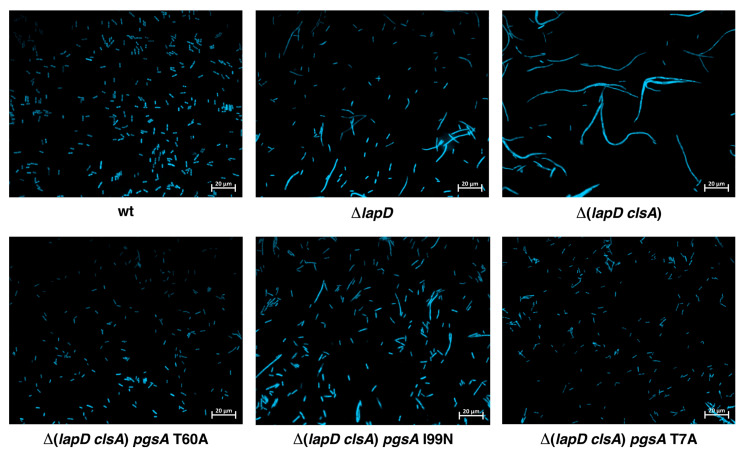
Loss-of-function suppressor mutations in the *pgsA* gene rescues the cell morphology defects of Δ(*lapD clsA*) bacteria. Microscopic images of the wild type, its isogenic Δ*lapD*, Δ(*lapD clsA*), and its derivatives with single-copy suppressor mutations in the *pgsA* gene. Bacterial cultures were grown overnight at 30 °C, diluted 1:100 in fresh LB, and grown until an OD_600_ of 0.6 at 30 °C. Cultures were harvested by centrifugation, and bacterial cells were stained with DAPI and imaged using fluorescence microscopy at ×1000 with a 20 µm scale. The relevant genotypes are indicated.

Fatty acid composition is known to control the cell length [40]. Increased fatty acid synthesis can lead to abnormal cell expansion, resulting in filamentous cell morphology. Δ*lapD* bacteria also exhibit cell morphology defects but are viable under normal laboratory growth conditions [14]. As Δ(*lapD clsA*) bacteria exhibit a sick phenotype even at 30 °C and have elevated PG levels with alterations in fatty acid composition, the cell morphology of such bacteria was examined in parallel with isogenic parental strains and derivatives with single amino acid substitutions that overcome their lethal phenotype. Exponentially grown bacteria were analyzed using fluorescence microscopy after staining with DAPI. Δ*lapD* bacteria exhibited a high proportion of filamentous cells. However, this phenotype was exacerbated in Δ(*lapD clsA*) bacteria (Figure 6). Most of the cells of Δ(*lapD clsA*) bacteria exhibited abnormally long cellular morphology. Quite significantly, suppressor mutations in the *pgsA* gene suppressed this severe long filamentous phenotype (Figure 6). The more robust suppressor mutation *pgsA* T60A virtually restored wild-type- like cell morphology. Notably, *pgsA* T7A also effectively suppressed the filamentous morphology of Δ(*lapD clsA*) bacteria (Figure 6). These data are consistent with the restoration of PL levels and fatty acid composition by such suppressor mutations. Taken together, the bacterial synthetic lethality of Δ(*lapD clsA*) and the sick phenotypes under permissive growth conditions are due to changes in fatty acid composition concomitant with severe cell morphology defects.

### 2.7. Suppressors of Δ(lapD clsA) Bacteria Do Not Alter LpxC Levels

**Figure 7:**
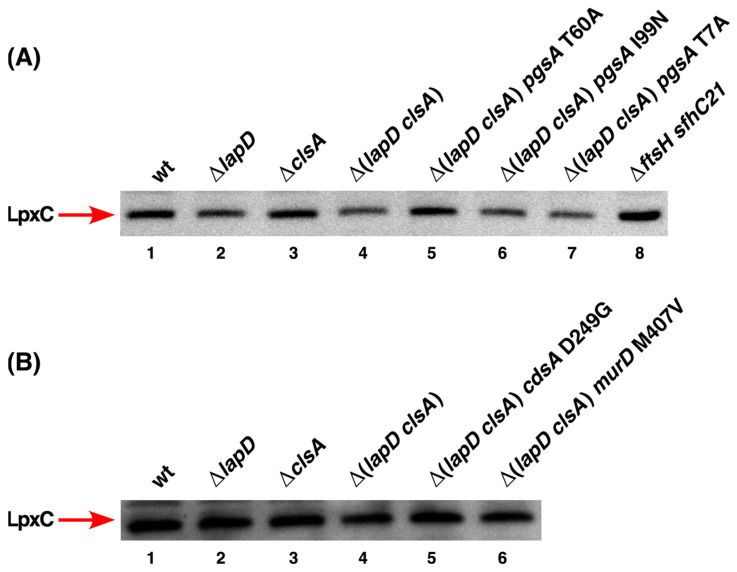
Suppression mutations in *pgsA*, *cdsA*, and *murD* genes do not act by altering LpxC levels. Immunoblots of whole-cell lysates obtained from isogenic strains grown at 30 °C in LB medium. An equivalent amount of total protein was resolved by 12% SDS-PAGE and transferred by Western blotting. Membranes were immunoblotted with LpxC-specific antibodies and revealed by chemiluminescence. Images from a representative experiment with the relevant genotypes are shown in panels (**A**,**B**). As an internal control, a cell lysate from the isogenic Δ*ftsH sfhC21* strain was applied ((**A**), lane 8). The arrow indicates the position of LpxC.

As PL and LPS biosynthesis utilize (*R*)-3-hydroxymyristate-ACP as a common metabolic precursor, it is possible that altered PL and fatty acid synthesis due to suppressor mutations in *pgsA* and *cdsA* might cause disturbances in LPS synthesis at the level of LpxC. LpxC is a key enzyme involved in regulating the balance between the LPS and PL biosynthetic pathways [4,5]. Thus, we examined LpxC levels using Western blotting. Isogenic cultures of wild type, Δ*lapD*, Δ*clsA*, Δ(*lapD clsA*), and its derivatives with suppressor mutations in *pgsA*, *cdsA*, and *murD* genes were grown under permissive growth conditions. As a control, cell lysates were prepared from isogenic Δ*ftsH sfhC21* bacteria that are known to have elevated levels of LpxC [4]. Equivalent amounts of proteins were transferred by Western blotting and immunoblotted with anti-LpxC antibodies. Estimation of LpxC levels revealed that none of the suppressor mutations in the *pgsA* gene caused any significant alteration in its amount (Figure 7, panel A). As expected, *ftsH* mutant bacteria showed increased LpxC levels. No impact of the *clsA* gene deletion was observed. Similarly, no significant changes in LpxC levels of Δ(*lapD clsA*) derivatives with the suppressor mutation in either *cdsA* or *murD* genes were observed (Figure 7, panel B). These results allow us to conclude that the single-copy extragenic suppressor mutation in either *pgsA* or *cdsA* or *murD* genes that overcome the lethal phenotype of the Δ(*lapD clsA*) combination do not operate by changing LpxC levels and are unlikely to impact LPS biosynthesis.

### 2.8. PgsA Co-Purifies with the Essential LapB Protein

**Figure 8:**
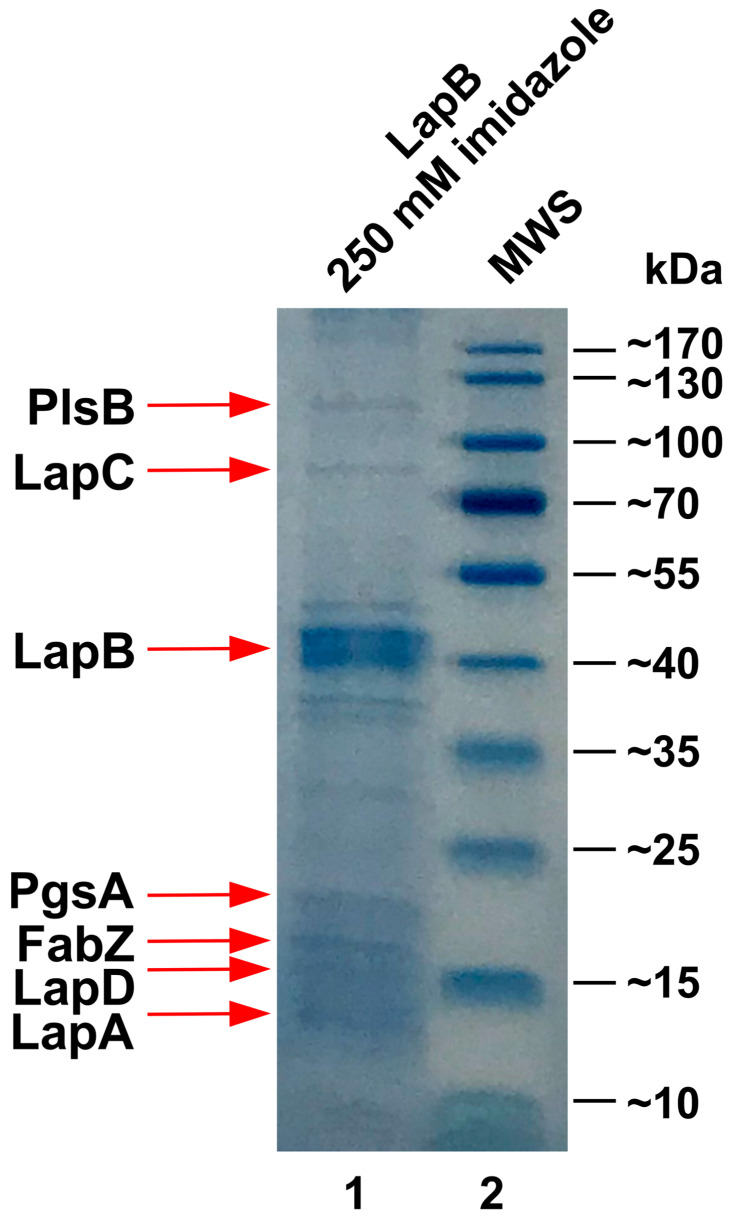
PgsA and PlsB are part of the LapB interactome. Purification profile of co-eluting proteins with His_6_-LapB (lane 1). Proteins were eluted using 250 mM imidazole. Proteins were resolved on a 12% SDS-PAGE. The identites of the co-eluting proteins are depicted by arrows.

Previously, we showed that the inner membrane essential protein LapD co-purifies with several proteins involved in LPS biosynthesis or its assembly (LapC, LapA, WaaC, and LapD) and the initiation of PL synthesis (FabZ) [5,8,13]. Hence, it was proposed that, in addition to regulating LpxC stability via interaction with FtsH, LapB could act as a scaffold for recruiting various enzymes in PL and LPS to coordinate their synthesis using its conserved tetratricopeptide repeats (TPR) [5]. Thus, we wondered whether any of the proteins examined in this study are also part of the LapB interactome. To achieve this, pull-down experiments were performed using the affinity purification of C-terminally His-tagged LapB, as described previously [5]. His-tagged LapB co-eluted with several proteins (Figure 8). MALDI-TOF analyses of co-purifying proteins revealed that the LapB complex, in addition to including known interacting partners such as LapA, LapC, LapD, and FabZ, also includes PgsA and PlsB (Figure 8). PlsB glycerol-3-phosphate acyltransferase catalyzes the first committed step in phospholipid biosynthesis [23]. Co-purification of PgsA and PlsB suggests that LapB-interacting partners are dedicated to PL and LPS biosynthesis.

### 2.9. Multicopy Suppressors of Δ(lapD clsA) Identify Factors That Can Inhibit Fatty Acid Biosynthesis or Regulate Cell Envelope Homeostasis

To further understand the physiological limiting factors that cause the lethality of Δ(*lapD clsA*) bacteria, multicopy suppressor analysis was performed. Two genomic plasmid DNA libraries were used. One of these was based on the ordered ASKA genomic library of cloned ORFs, where the expression of individual genes is under the control of an inducible, tightly regulated P_T5_-*lac* promoter [41]. Transformants of Δ(*lapD clsA*) with this library were selected at 42 °C in the presence of 75 μM IPTG. The second library was generated using the mini-Mu system [42]. In this case, the first deletion derivatives of *clsA* and *lapD* genes were transduced into the p15A mini-Mu lysogenic strain, and lysates prepared from such strain were used to generate plasmid libraries. Transductants in the Δ(*lapD clsA*) background were plated to isolate Ts^+^ colonies. Plasmid DNAs from such Ts^+^ strains were isolated, verified by retransformation, and used to identify the minimal coding sequence by DNA sequencing that could restore the growth of Δ(*lapD clsA*) bacteria. The usage of mini-Mu-based libraries was necessitated to prevent recloning of *lapD* and *clsA* genes, which was the case with the ASKA library. These multicopy suppressors identified a set of genes involved in fatty acid/PL synthesis or regulation of cell envelope-related functions (Table 4).

**Figure 9:**
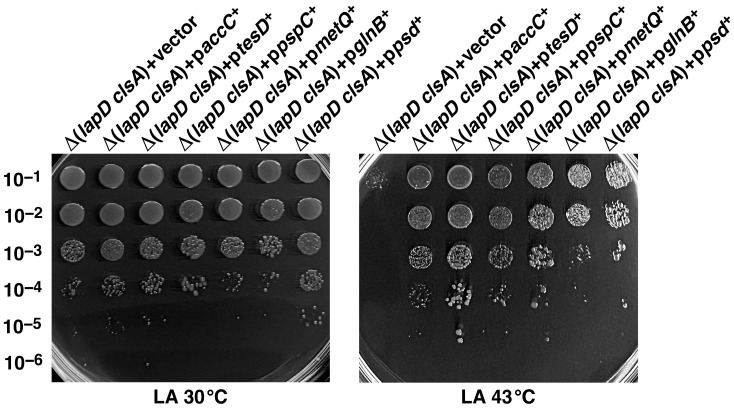
Overexpression of specific multicopy suppressors that can reduce fatty acid biosynthesis (*accC*, *tesD*, and *glnB*) can overcome the synthetic lethality of Δ(*lapD clsA*) bacteria. Growth of isogenic cultures of Δ(*lapD clsA)* with the vector alone or its isogenic derivatives carrying plasmids expressing various multicopy suppressors from the P_T5_-*lac* promoter was quantified using spot dilution on LA at 30 °C and LA media supplemented with 75 μM IPTG at 43 °C. The plates were incubated for 24 h. Data from a representative experiment with the indicated genotypes are shown.

To validate these results, the suppression ability was analyzed using a spot-dilution assay of Δ(*lapD clsA*) derivatives carrying these genes expressed from the P_T5_-*lac* promoter (Figure 9). These results revealed a variable degree of restoration of bacterial growth at 43 °C in the presence of 75 μM IPTG, although all the analyzed strains performed better than the parental strain with the vector alone. The most robust suppression was observed with the gene encoding putative thioesterase *tesD*, followed by *pspC* and *accC* genes when mildly overexpressed (Figure 9). Noticeable restoration of bacterial growth at 43 °C was also observed when *glnB*, *psd*, and *metQ* genes were overexpressed.

The products of some genes can inhibit fatty acid biosynthesis or affect PL biosynthesis. The most prominent of these was the repeated cloning of the *accC* gene, which encodes the biotin carboxyl carrier protein, one of the four subunits of the ACC complex [21]. AccC catalyzes the first step of the acetyl-CoA carboxylase reaction mediated by the ACC enzyme. It has been demonstrated that for the enzymatic activity of the ACC complex, all four subunits must be maintained in stoichiometric amounts, and any imbalance can inhibit the ACC enzymatic activity [14]. These results are consistent with the demonstration of a reduction in the amount of PL when one of the subunits of the ACC complex is overproduced or mutated [14]. Another interesting multicopy suppressor identified is the *glnB* gene (Table 4). The *glnB* gene encodes the PII-1 protein, which is involved in the regulation of nitrogen metabolism by controlling the activity of glutamine synthetase [43,44,45]. However, more importantly, in the context of this study, GlnB also acts as a regulator of fatty acid synthesis via its interaction with the biotin carboxylase/biotin carboxyl carrier protein (BC-BCCP) subcomplex of the ACC enzyme [46]. GlnB interacts with BC-BCCP to form a ternary complex that inhibits the ACC enzyme activity [46]. Another gene whose overexpression rescues the lethality of Δ(*lapD clsA*) is the *psd* gene, encoding phosphatidylserine decarboxylase. Psd catalyzes the formation of the most abundant PL species, phosphatidylethanolamine. The isolation of PspC as a dosage-dependent suppressor can be rationalized, as it has been shown to be a substrate of FtsH protease [47]. MetQ is a lipoprotein and its overproduction could cause diversion of PG pools that can overcome toxic accumulation of PG and thereby overcome lethality. Thus, multicopy suppressor analysis further reinforces our results that LapD and ClsA absence leads to dysfunctional fatty acid/PL biosynthesis, causing an increase in PG amounts. Currently, the precise function of TesD is unknown; however, its predicted polypeptide has a characteristic hot-dog fold observed in the family of thioesterases and could act by hydrolyzing acyl chains.

### 2.10. Overexpression of accC and tesD Suppresses Elevated Accumulation of PL Species in Δ(lapD clsA) Bacteria

**Figure 10:**
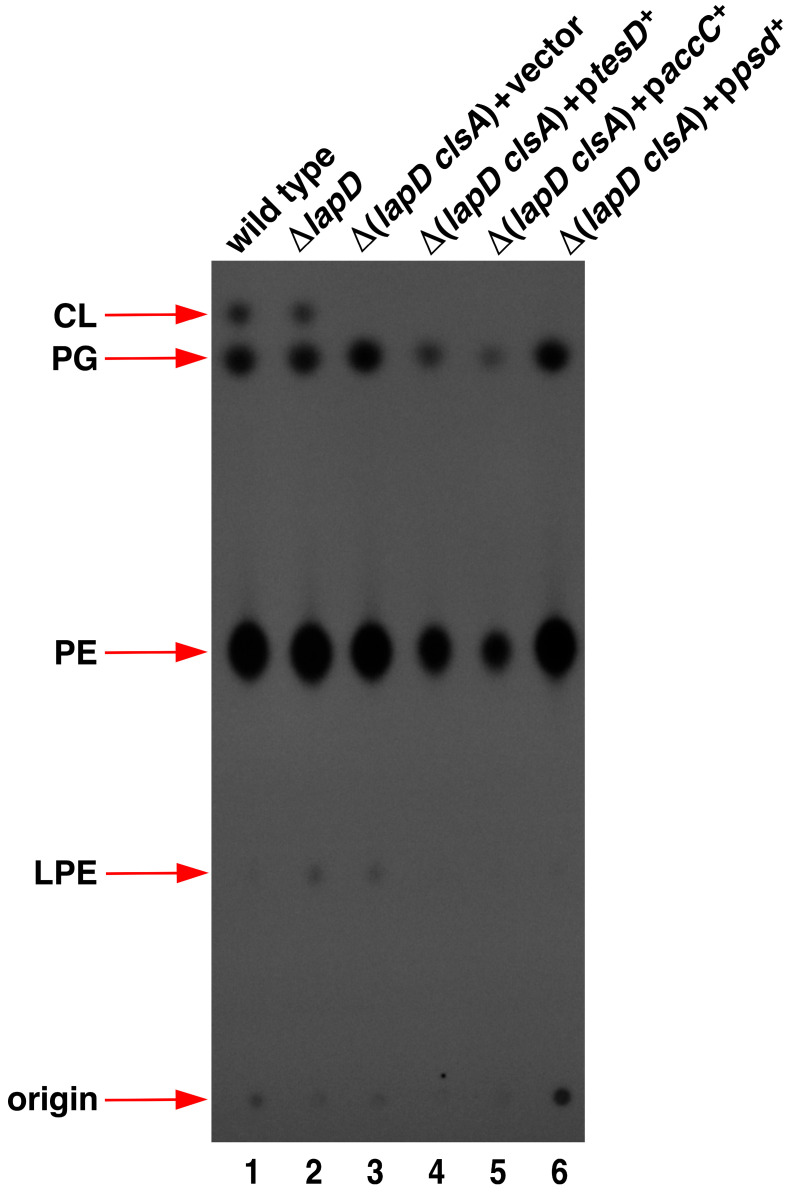
Overexpression of either *tesD* or *accC* genes in Δ(*lapD clsA*) can overcome their synthetic lethality by decreasing phospholipid synthesis. Thin-layer chromatography of ^32^P-labeled PLs extracted obtained from isogenic wild type, Δ*lapD*, Δ(*lapD clsA*) with empty vector and its derivatives carrying plasmids expressing either t*esD* or *accC* or *psd* genes from P_T5_-*lac* promoter. A representative image from three biological replicates is shown. The arrows indicate the positions of major PL species.

To address the molecular basis of the restoration of growth of Δ(*lapD clsA*) bacteria by overexpression of various multicopy suppressor-encoding genes, PL analysis was undertaken using TLC. We specifically analyzed those, which are predicted to inhibit fatty acid biosynthesis and alter PL composition. Thus, isogenic cultures of derivatives of Δ(*lapD clsA*) with the vector alone or expressing plasmid-born *accC*, *psd*, and *tesD* were labelled with inorganic phosphate in the presence of 75 μM IPTG to induce gene expression. Samples from such cultures were used to extract PLs and analyzed for their composition using TLC. As is evident, Δ(*lapD clsA*) had more than 33% elevation of PG amounts compared to the parental wild type, which was significantly suppressed by overexpression of either *tesD* or *accC* gene (Figure 10, lanes 3 vs. 4 and lanes 3 vs. 5). Densitometric quantification of PL species when the *tesD* gene was overexpressed showed PG to be approximately 47% and PE nearly 63% compared to the parental wild type, which was taken as 100%. An even more severe reduction in PG and PE amounts was observed when the *accC* gene was overexpressed. Thus, the PG amounts were approximately 24% and PE only 46% compared to the parental wild type upon *accC* overexpression. As mentioned above, overproduction of any of the subunits of the ACC complex can disturb the balance in the stoichiometry of various components, leading to the inhibition of ACC enzymatic activity that catalyzes the first committed step in the initiation of fatty acid synthesis. If TesD is proven to be a thioesterase, it could impact by releasing acyl chains by hydrolysis that can reduce PL synthesis. No significant alterations were observed with overexpression of the *psd* gene, consistent with the observation of homeostatic control of fatty acid biosynthetic enzymes and the lack of changes in PL composition due to the amplification of Psd enzymes [48].

### 2.11. Overexpression of pspC Elevates LpxC Levels

**Figure 11:**
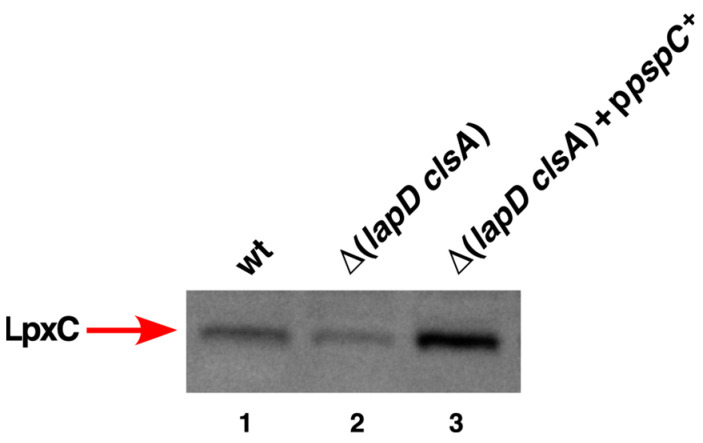
Overexpression of the *pspC* gene in Δ(*lapD clsA*) stabilizes LpxC. Immunoblot of whole cell lysates obtained from isogenic wild type, Δ(*lapD clsA*) with empty vector, and its derivative carrying plasmid expressing the *pspC g*ene from P_T5_-*lac* promoter. Cultures were grown at 30 °C and shifted to 42 °C until an OD_595_ of 0.6. An equivalent amount of total protein was resolved on a 12% SDS-PAGE and transferred by Western blotting. Membranes were immunoblotted with LpxC-specific antibodies and revealed by chemiluminescence. Images from a representative experiment with the relevant genotypes are shown.

One of the multicopy suppressors that restores the growth of Δ(*lapD clsA*) at high temperatures is the *pspC* gene. PspC is a positive regulator of the *psp* operon and has been suggested to be a substrate of FtsH protease [47]. The FtsH-LapB complex catalyzes the proteolysis of LpxC. Thus, we rationalized that excess of PspC could titrate FtsH, which could potentially stabilize LpxC. To test this hypothesis, we examined LpxC levels using immunoblotting. Total cell lysates were prepared from isogenic wild type, Δ(*lapD clsA*), and its derivative expressing the *pspC* gene under the control of the inducible P_T5_-*lac* promoter and analyzed by Western blotting. Estimation of LpxC levels revealed that overexpression of the *pspC* gene led to an increase in LpxC amounts compared to those observed in the parental wild type and its Δ(*lapD clsA*) derivative (Figure 11). These results provide a rationale explanation for the isolation of *pspC* gene as a multicopy suppressor of the Ts phenotype of Δ(*lapD clsA*) bacteria by stabilization of LpxC.

## 3. Discussion

The outer membrane of Gram-negative bacteria is primarily composed of lipopolysaccharides and phospholipids. In *E*. *coli*, FASII generates two products, acyl-ACP and β-hydroxyacyl-ACP, which are components of LPS and PLs biosynthesis. Acyl-ACPs are used by PlsB and PlsC enzymes to generate phosphatidic acid, which is a precursor of all cytoplasmic membrane phospho- and glycolipids [3]. β-Hydroxyacyl-ACP molecules serve as substrates for the acyltransferases catalyzing the initial steps in the biosynthesis of the essential conserved lipid A component of LPS. We previously showed that LapD is one of the factors that links the LPS and PL biosynthetic pathways, as it co-purifies with various components of PL and LPS biosynthesis [13]. Genetic and biochemical studies have shown that although LapD is non-essential for bacterial growth, it becomes indispensable when the lipid A is underacylated (absence of LpxM myristoyl acyltransferase) or when LPS is composed of only Kdo_2_-lipid A due to the lack of heptosyltransferase WaaC [13,14]. We showed that suppressors that bypass the lethality of Δ(*lapD lpxM*) and Δ(*lapD waaC*) map to either various subunits of the essential enzyme acetyl-CoA carboxylase transferase or components of LPS transport and peptidoglycan biosynthesis [14]. Here, we show that LapD also becomes conditionally essential in the absence of the major cardiolipin biosynthesis pathway. CLs are the only PLs that are non-essential for bacterial viability. Thus, it is intriguing that cardiolipins are required for bacterial viability in the absence of LapD. However, CL synthesis has been shown to be required for viability of bacteria lacking LpxM [18,19]. Although the Δ(*lapD clsA*) mutational combination has been shown to confer lethality, its molecular basis has not been addressed [13,49]. In this study, we showed that Δ(*lapD clsA*) bacteria exhibited severe morphological defects. We used this conditional synthetic lethal phenotype to isolate extragenic chromosomal and multicopy suppressors to investigate its molecular basis.

Characterization of these suppressors showed that the majority of single-copy suppressor mutations mapped to the *pgsA* gene, encoding phosphatidylglycerophosphate synthase, whose product is required for PG synthesis (Figure 1). PG synthesis occurs via the condensation of CDP-DAG with G3P mediated by PgsA to form the intermediate PG phosphate, which is then dephosphorylated by Pgp phosphatase to form PG [50]. Another set of suppressor mutations was mapped to the *cdsA* gene encoding CDP-diglyceride synthetase. CDP-DAG is the central precursor of all PL in *E*. *coli*. Interestingly, the most frequent single amino acid exchange observed in the Ts^+^ suppressors of Δ(*lapD clsA*) was in the amino acid residue Thr 60 in PgsA. Alteration of this highly conserved amino acid residue impairs PgsA activity [36,51]. To our knowledge, this is the first study to identify suppressor mutations in the *pgsA* gene in the absence of LapD. Analysis of the PL content of Δ(*lapD clsA*) bacteria revealed elevated levels of PG accumulation, which can contribute to the toxicity and severe defects in cell division observed in these bacteria. Importantly, loss-of-function *pgsA* suppressor mutations restored the wild-type-like cell morphology and significantly reduced PG abundance. Notably, suppressor mutations in the *pgsA* gene were shown to repress highly elevated PL levels, particularly in the presence of the PgsA T60A amino acid exchange. Thus, Δ*lapD* bacteria are sensitized due to an imbalance in either PL composition, or underacylation of lipid A, or when LPS is composed of only Kdo_2_-lipid A. While extragenic suppressors of Δ(*lpxM clsA*) were mapped to the *msbA* gene encoding LPS flippase [16], suppressors of Δ(*lapD clsA*) were mapped to PL biosynthetic genes, implying that CLs have a dual function. Thus, CLs can assist MsbA in LPS translocation and cooperate with LapD to maintain a balanced fatty acid composition.

Acidic phospholipids, including CL, localize to cell poles and interact with key players such as RodZ [52,53]. The absence of CL in Δ*lapD* could be one of the reasons for the severe cell morphology defects in Δ(*lapD clsA*) bacteria. Consistent with this notion, *rodZ* has been shown to be essential in the absence of LapD, although no explanation for this lethality has been provided [54]. Furthermore, it is well established that *E. coli* size is dependent on fatty acid/PL levels [40,55,56]. The suppression of cell morphology defects of Δ(*lapD clsA*) bacteria by loss-of-function mutations in the *pgsA* gene is consistent with the prominent role of anionic PLs in cell morphology. Anionic lipids have been shown to be required for the assembly of the Z-ring and for DNA replication [57,58,59]. In this study, we observed an abnormally high accumulation of PG in Δ(*lapD clsA*) bacteria, which was relieved by *pgsA* suppressor mutations. Consistent with these results, we previously showed restoration of normal cell morphology when fatty acid biosynthesis was repressed by overexpression of either the *tesA*’ thioesterase gene, induction of *acpP*, or inhibition of ACC complex activity [14].

Interestingly, gross alterations in Δ(*lapD clsA*) bacteria were observed in FFA composition with an increased presence of saturated vs. unsaturated fatty acids, which was particularly reflected by a 3-fold increase in 18:0 and a 20–40% decrease in the presence of *cis*-vaccenic acid (18:1). Increased accumulation of *cis*-vaccenic unsaturated fatty acid can lead to a decrease in LPS biosynthesis, which can reset the balance between LPS and PL amounts, as observed in Δ(*lapD lpxM*) bacteria [14]. Significantly, *cis*-vaccenic acid amounts were restored in the presence of suppressor mutations in either *cdsA* or *pgsA* genes. Overall, these results imply that the Δ(*lapD clsA*) lethal phenotype stems from alterations in the fatty acid and PL composition. Another interesting suppressor mutation was mapped to the essential *murD* gene, which connects PGN synthesis and LapD function, consistent with our earlier findings [14].

In support of the results revealing excess PG and changes in fatty acid composition, the multicopy suppressor approach revealed that reducing fatty acid synthesis by inhibiting acetyl-CoA carboxylase activity, which catalyzes the rate-limiting step in the initiation of fatty acid synthesis, can overcome the lethality at elevated temperatures. This approach revealed that overexpression of either *accC*, encoding the subunit of the ACC enzyme, or *glnB* effectively restored the growth of Δ(*lapD clsA*) bacteria. The ACC complex has four subunits, and all components are held in stoichiometric amounts [60]. Overexpression of any of the subunits reduces PL and fatty acid content by decreasing the ACC enzyme activity [14]. We showed a significant decrease in PG molecules, which are otherwise elevated in Δ(*lapD clsA*) bacteria when the AccC subunit is overproduced, providing a genetic and biochemical link to the reasons for the lethality and mode of growth restoration of Δ(*lapD clsA*) bacteria. Another multicopy suppressor is encoded by the *glnB* gene, whose product belongs to the PII signal transduction family and plays a key role in nitrogen metabolism [43,44,45]. However, GlnB proteins from Proteobacteria have also been shown to interact with the biotin carboxyl carrier protein (BCCP) of acetyl-CoA carboxylase (ACC) [46,61]. In *E. coli*, this GlnB interaction reduces the k_cat_ of acetyl-CoA carboxylation [46]. This overproduction of GlnB should result in the reduced ACC enzyme activity, which can provide a rational explanation for its role as a dosage-dependent suppressor. The isolation of *metQ* as a multicopy suppressor is interesting because the encoded protein is a predicted lipoprotein that is part of the methionine transport system. Prosite annotation suggests lipidation at amino acid position 23, which could be a PG moiety. MetQ is included in the TIGRFAM lipoprotein TIGR00363/NlpI family (http://ca.expasy.org/prosite (accessed on 9 October 2025)). PspC, whose overproduction also suppresses the Ts phenotype, acts as a positive regulator of the *psp* operon [62]. We speculate that overproduction of MetQ could titrate out PG molecules, which are present in toxic amounts in Δ(*lapD clsA*) bacteria. However, MetQ is not well characterized, and further studies are required to validate its lipidation and its impact on PL pools. However, it has parallels, as the absence of the most abundant lipoprotein, Lpp, allows the growth of *pgsA* mutant bacteria [63]. The rationale for Δ*lpp* to relive the lethality of *pgsA* mutants is based on the known PG attachment to this highly abundant lipoprotein, which can lead to the availability of PG molecules that are very limiting in *pgsA* mutant bacteria [64]. The isolation of *pspC* as a multicopy suppressor is interesting and can be explained by the observed stabilization of LpxC upon its overexpression in Δ(*lapD clsA*) bacteria. This is consistent with PspC being a substrate of FtsH [47]. We explain these results because titration of FtsH by excess of PspC can lead to the stabilization of LpxC. This can reset the LPS vs. PL balance, which is disturbed in Δ(*lapD clsA*) bacteria. These results are further supported by our previous findings showing that LpxC stabilization can overcome the growth defects of Δ*lapD* bacteria [13]. Thus, increasing either the stability of LpxC or repressing PL synthesis can overcome the lethality of Δ(*lapD clsA*) bacteria, positioning LapD as the link between LPS and FA synthesis. Another possibility of PspC-mediated suppression is the association of CL with Psp proteins in polar membrane regions, which can affect interactions with the RodZ/MreB cytoskeleton complex and Tat-dependent protein translocation [65]. It is possible that PspC is limiting in Δ(*lapD clsA*) bacteria, which can impact the extracytoplasmic stress response, which is elevated in the absence of LapD [13,14].

Similarly, we can explain the isolation of putative thioesterase TesD, as its overproduction reduced PL synthesis and restored growth at elevated temperatures. We have not yet biochemically established the thioesterase activity of TesD, and this awaits confirmation. However, TesD has a characteristic hot-dog fold, which is present in the thioesterase superfamily [66]. Thioesterases typically catalyze the cleavage of thioester bonds in a wide range of activated fatty acyl coenzyme A (CoA) substrates, acyl carrier proteins (ACPs), and other cellular molecules [66]. Importantly, it has been shown that thioesterase overproduction can alter the degree of saturation of the membrane lipids in *E. coli* [67]. It has been shown that the biosynthetic coupling between fatty acid and phospholipid syntheses could be disrupted by the high-level expression of thioesterases [68]. The isolation of *tesD* is also supported by our earlier results with overexpression in the cytoplasm of *tesA*’ lacking its signal sequence, which effectively suppressed PL accumulation in Δ*lapD* derivatives [14]. Isolation of *psd* as a multicopy suppressor is also logical, as its product encodes phosphatidylserine decarboxylase catalyzing the synthesis of PE.

Thus, based on our results of the synthetic lethality of the concomitant absence of LapD and CL, we can conclude that they together constitute essential components required to maintain the homeostasis of essential cell envelope components. In such bacteria, several key components, particularly the composition of PLs, fatty acids, and cellular morphology, are severely affected. Thus, Δ(*lapD clsA*) bacteria accumulated toxic amounts of PG species of PLs, causing gross alterations in the fatty acid composition, which resulted in defects in cellular morphology. Our results show that lethality can be overcome by reducing PG synthesis, as shown by the preponderance of suppressor mutations that relieve this lethality mapping to the *pgsA* gene. Furthermore, Δ(*lapD clsA*) bacteria accumulated less unsaturated fatty acids, particularly *cis*-vaccenic acid, with a significant increase in saturated fatty acids. This imbalance can cause alterations in the regulation of membrane fluidity and hence, Δ(*lapD clsA*) bacteria cannot withstand temperatures above 37 °C, which contributes to the synthetic lethal phenotype. Suppressor mutations in the *pgsA* gene, which are recessive, not only reduced the hyperelevated toxic accumulation of PG but also corrected cellular morphological defects. These findings were supported by the isolation of multicopy suppressors, whose overexpression can inhibit the initiation of fatty acid at the level of the acetyl coenzyme A carboxylase enzyme. Notably, we identified a new putative thioesterase, TesD, whose overproduction effectively prevents the synthetic lethality of Δ(*lapD clsA*) bacteria and significantly reduces the accumulation of elevated PL species. Currently, efforts are underway to establish the thioesterase activity of TesD and its specificity for the acyl chain length. Thus, we can conclude that the synthetic lethality of Δ(*lapD clsA*) is due to the increased toxic accumulation of PG species and an altered ratio of saturated and unsaturated fatty acids. Hence, consistent with our present results and previous findings, LapD is a multitasking protein that connects LPS, PL, and PGN synthesis, and cardiolipins play a pivotal role in this process. This is further supported by the finding that PgsA is part of the large protein complex of LapB/LapD, which can lead to the coordination of LPS and PL biosynthetic pathways.

## 4. Materials and Methods

### 4.1. Bacterial Strains, Plasmids and Media

The bacterial strains and plasmids used in this study are described in Table 5.

Luria–Bertani (LB) broth and agar (LA) (Difco, Franklin Lakes, NJ, USA) were prepared as described earlier [75]. As per the experimental requirements, the media were supplemented with ampicillin (100 μg/mL), kanamycin (50 μg/mL), tetracycline (10 μg/mL), or chloramphenicol (20 or 30 μg/mL). The expression of various genes in the pCA24N expression vector was induced by adding 75 μM IPTG to the growth medium.

### 4.2. Strain Construction and Isolation of Extragenic Chromosomal Suppressors

All strains used in this study were derived from W3110, serving as the parental wild-type strain, unless otherwise stated. The construction of a non-polar antibiotic-free deletion derivative of *lapD* SR25204 in W3110 has been previously described [14]. Bacteriophage P1-mediated transductions were performed as previously described [76]. The Δ*lapD* derivative SR25204 served further as a recipient to construct the Δ(*lapD clsA*) derivative SR25209 by bacteriophage P1-mediated transduction. To construct this strain, the Δ*clsA* non-polar deletion was first constructed by replacing the coding sequence of the *clsA* gene by the *aph* cassette from the pKD13 plasmid using the λ recombinase system, as described [17]. The *aph* antibiotic cassette was removed by transformation with pCP20 plasmid DNA, which expresses a site-specific recombination flippase [72]. To construct Δ(*lapD clsA*), bacteriophage P1-mediated transductions were performed in reciprocal order and plated on LA agar plates at 30 °C, which was found to be a permissive condition for such derivatives. As a control, each recipient in the transduction experiments was transformed with a covering plasmid expressing either *lapD* or *clsA* genes. To isolate extragenic chromosomal suppressor mutants, cultures of several independently obtained suppressor-free Δ(*lapD clsA*) transductants grown at 30 °C were plated at 42 °C. The Ts^+^ properties were verified by re-streaking. Cultures from these Δ(*lapD clsA*) Ts^+^ isolates, 65 putative suppressor-containing strains, were retained. Suppressor mutations were marked with mini-Tn*10* Tet [77], and verified that the Tn*10*-linked suppressor mutation breeds true. To identify the suppressor mutation, linked Tn*10* mutations were recombined onto single-copy cosmid clones using a previously described DNA library [14,74]. To identify the candidate gene with a suppressor mutation, DNA from recombinant cosmids was subcloned, selecting for Tn*10* Tet and Amp markers or by inverse PCR using chromosomal DNA to identify Tn*10* junctions, as described [78]. DNA from the recombinant plasmid was used to sequence the Tn*10* ends and flanking regions using inverse PCR, as previously described [8,78]. This allowed us to place 14 suppressor mutations in three complementation groups. The same cosmid DNAs were then used to sequence the candidate genes using specific oligonucleotides.

### 4.3. Identification of Multicopy Suppressors, Whose Overexpression Overcomes Lethal Phenotype at 42 °C

DNA from plasmid pools obtained from the ordered genomic library cloned in pCA24N carrying all predicted ORFs of *E. coli* (ASKA collection) [41] was used to transform the Δ(*lapD clsA*) strain SR25209, selecting directly for Ts^+^ survivors at 42 °C in the presence of 75 μM IPTG. Cultures for Ts^+^ colonies were grown to isolate plasmid DNAs, which were used to retransform the Δ(*lapD clsA*) strain SR25209 for the validation of growth restoration at 42 °C. The identity of the cloned gene, whose overexpression conferred suppressing ability, was obtained by DNA sequencing. However, this method gave a lot of background due to the recloning of *lapD* and *clsA* genes, although several *bona fide* multicopy suppressors were also isolated. To overcome this drawback, the mini-Mu in vivo cloning approach was used [42,70]. To achieve this, a Δ(*lapD clsA*) derivative was constructed in a derivative with Muc ts62 Mud5005, resulting in the construction of SR23769. Mini-Mu lysates of SR23769 were prepared by thermal induction and used to transduce plasmids into Δ(*lapD clsA*) derivative SR25209, selecting for Ts^+^ colonies at 42 °C. Plasmid DNAs from such Ts^+^ derivatives were subcloned into a p15A-based medium copy plasmid vector pMBL18 [73] to identify the minimal coding region that could suppress the lethality of Δ(*lapD clsA*) at 42 °C. Plasmids that bred true in suppressing ability were used for DNA sequencing.

### 4.4. Isolation and Analysis of ^32^P-Labeled Phospholipid Species

Overnight cultures of isogenic strains of wild type, Δ*lapD*, Δ(*lapD clsA*), and its derivatives with suppressor mutations in *pgsA*, *cdsA*, and *murD* were grown in LB medium under permissive growth conditions at 30 °C. The cultures were diluted, adjusted to an OD_595_ of 0.05, and grown for another 45 min and labelled with 2.5 μCi/mL of ^32^P_i_ in 2.5 mL of LB medium until an OD_595_ of 1.0. For the growth of strains Δ(*lapD clsA*) derivatives carrying multicopy suppressors, during the labelling period, 75 μM IPTG was added for the expression of P_T5_-*lac* promoter-inducible *accC*, *psd*, and *tesD* genes. Cultures were harvested by centrifugation at 4300× *g* for 10 min. The pellets were dispersed in water. Phospholipids were extracted using the procedure described by Bligh and Dyer, and Ames [79,80]. The PLs were collected from the lower organic phase and dried under a stream of nitrogen. Dried samples containing phospholipids were reconstituted in 50 μL of a 2:1 chloroform/methanol mixture. Before the extraction of PLs, an aliquot of the samples was used to measure the total protein concentration using a Pierce BCA protein assay kit (ThermoScientific, Warsaw, Poland). Equivalent amounts (10,000 cpm/lane) of ^32^P-labeled phospholipids after adjusting for protein concentration were separated on TLC Silica gel 60 F254, aluminum sheets, 20 cm × 20 cm plates (Merck, Mississauga, ON, Canada). Lipids were separated using a solvent system, as described [14]. The TLC plates were dried and exposed to CL-Xposure^Tm^ Film (ThermoScientific, Rockford, IL, USA) or exposed to a PhosphorImager screen to visualize and quantify the labeled PLs using the Amersham Typhoon Biomolecular Imager (Cytiva, Uppsala, Sweden). Densitometry analysis of the autoradiograms was performed using the ImageJ software (rsb.info.nih.gov). Three biological replicates were used for quantification.

### 4.5. Isolation of Fatty Acids and Their Analysis Using Gas Chromatography

Isogenic bacterial cultures were grown at 28 and 37 °C as described for phospholipid extraction, without adding either IPTG or ^32^P_i_. Fifteen mL cultures at an OD_595_ of 1.0 were harvested by centrifugation at 4300× *g* for 10 min, and the pellets were frozen. The pellets were thoroughly resuspended in 2.5 mL of H_2_O with vortexing, followed by the addition of 5 μL of 10 mg/mL heptadecanoic acid (C17:0) (Merck, Warsaw, Poland) dissolved in ethanol, as an internal standard. Free fatty acids were extracted as per the procedure described [14,81]. Fatty acid methyl esters were obtained by treating the dried extract with 0.5 mL of 1.25 M HCl in methanol and incubating at 50 °C for 15 h. Fatty acids were extracted twice in 500 μL of hexane. FFA were analyzed by gas chromatography using a Shimadzu GC-2010 Pro (Shimadzu, Kyoto, Japan) with a 30 m × 0.25 mm capillary column under the experimental conditions described [14]. One µL of each sample was injected using a 1:100 split ratio of the helium carrier gas. For calibration, a fatty acid methyl ester mixture C8-C22 (CRM18920 Merck, Warsaw, Poland) was used to establish the retention time of various peaks corresponding to different fatty acids. Each experiment was repeated with three independent biological repeats. The data were analyzed using the software provided by Shimadzu (Kyoto, Japan).

### 4.6. Estimation of LpxC Amounts by Immunoblotting

Isogenic strains of wild type, Δ(*lapD clsA*), and its derivatives with either chromosomal extragenic suppressor mutation or carrying plasmid born multicopy suppressors were routinely grown at 30 °C in 5 mL LB medium up to an OD_595_ of 1.0. The cultures were harvested by centrifugation at 7000× *g* for 10 min. The cell pellets were resuspended in SDS lysis buffer. As an internal control, cell lysates were prepared from the Δ*ftsH sfhC21* derivative grown under similar conditions. Protein concentrations were measured using the Pierce BCA protein assay kit (ThermoScientific, Warsaw, Poland). An equivalent amount of protein was applied to a 12% SDS-PAGE for resolution. After electrophoresis, the proteins were blotted to a PVDF membrane. Membranes were immunoblotted with LpxC-specific antibodies and revealed using a chemiluminescence kit (ThermoScientific, Warsaw, Poland) according to the manufacturer’s instructions.

### 4.7. Examination of Cellular Morphology and Measurement of Bacterial Growth

For quantification of bacterial growth, isogenic bacterial cultures were grown overnight in LB medium at 30 °C. Exponentially grown cultures were adjusted to an OD_595_ of 0.1, serially diluted, and bacterial growth was measured using spot-dilution assay as described [82]. To examine cellular morphology, cultures of wild type, Δ*lapD*, Δ(*lapD clsA*), and its three isogenic derivatives with independent chromosomal suppressor mutations in the *pgsA* gene were grown in LB medium. Overnight cultures were diluted 1:100 and allowed to grow to an OD_595_ of 0.6 under permissive conditions at 30 °C in LB medium. Aliquots of cultures were centrifuged at 4300× *g* for 5 min and incubated for 10 min in TBS supplemented with 10 µg/mL of 4′,6-diamidino-2-phenylindole (DAPI) stain. Samples were immobilized on agarose pads, and cell morphology was examined using epifluorescence and differential interference contrast (DIC) microscopy, as described [14]. Cellular morphology was observed using a Zeiss apotome microscope (Carl Zeiss, Jena, Germany).

### 4.8. Purification of LapB

The production of C-terminal His_6_-tagged LapB was induced with 0.3 mM IPTG, using expression system as described [5]. Inner membrane proteins were extracted in binding buffer containing 50 mM NaH_2_PO_4_, 300 mM NaCl, 10 mM imidazole (buffer A) supplemented with 1% octyl-β-D-glucoside. After centrifugation, the supernatant was purified by FPLC (AKTA Pure, Cytiva, Warsaw, Poland) using a 1 mL HisTrap FF column. The column was washed with a binding buffer containing 20 mM imidazole to remove non-tagged proteins. Proteins were eluted with buffer A using a step gradient ranging from 50, 100, 250, and 500 mM imidazole and analyzed on 12% SDS-PAGE.

## Figures and Tables

**Table 1:** Δ(*lapD clsA*) bacteria exhibit synthetic lethality at 42 °C.

Genotype of Recipient	Number of Transductants					
	P1 Δ*clsA*			P1 Δ*lapD*		
	30 °C	37 °C	42 °C	30 °C	37 °C	42 °C
wt	1290	1363	1180	1406	1512	955
Δ*lapD*	633	455 ^a^	6	ND	ND	ND
Δ*lapD* + p*clsA*^+^	1234	1340	1185	ND	ND	ND
Δ*clsA*	ND	ND	ND	430	84 ^a^	3
Δ*clsA* + p*lapD*^+^	ND	ND	ND	1293	1365	1196

^a^ small colonies, ND denotes not determined.

**Table 2:** Mapping of extragenic chromosomal suppressors of Δ(*lapD clsA*) bacteria.

Single-Copy Chromosomal Suppressors of Δ(*lapD clsA*)			
Gene	Function	Mutation Position	Number of Isolates
*pgsA*	phosphatidylglycerophosphate synthase	T60A (ACC → GCC)	4
		T60P (ACC → CCC)	1
		T7A (ACG → GCG)	3
		I99N (ATC → AAC)	2
*cdsA*	CDP-diglyceride synthetase	D249G (GAC → GGC)	3
*murD*	UDP-*N*-acetylmuramoyl-L-alanine:D-glutamate ligase	M407V (ATG → GCG)	1

**Table 3:** Measurement of relative fatty acid composition using gas chromatography. The data are presented for three biological replicates grown at 28 and 37 °C, and the average with S.E is shown.

**LB 28 °C**								
**Fatty Acid Species**	**wt**	**Δ*lapD***	**Δ*clsA***	**Δ(*lapD clsA*)**	**Δ(*lapD clsA*)** ***pgsA*** **T60A**	**Δ** **(*lapD clsA*)** ***pgsA*** **I99N**	**Δ(*lapD clsA*)** ***pgsA*** **T7A**	**Δ(*lapD*** ***clsA*** **)** ***cdsA*** **D249G**
12:0	0.15 ± 0.01	0.33 ± 0.02	0.24 ± 0.01	0.18 ± 0.01	0.16 ± 0.02	0.19 ± 0.01	0.17 ± 0.01	0.241 ± 0.01
14:0	2.61 ± 0.03	2.73 ± 0.03	3.19 ± 0.04	2.51 ± 0.02	1.52 ± 0.01	3.36 ± 0.03	2.36 ± 0.01	2.16 ± 0.02
16:0	34.80 ± 0.07	38.44 ± 0.09	36.81 ± 0.17	38.48 ± 0.12	37.36 ± 0.20	37.43 ± 0.23	38.08 ± 0.29	38.77 ± 0.31
18:0	0.75 ± 0.02	1.16 ± 0.01	0.54 ± 0.01	2.30 ± 0.03	1.34 ± 0.03	2.63 ± 0.04	0.86 ± 0.01	0.91 ± 0.02
16:1	31.89 ± 0.31	31.49 ± 0.26	34.45 ± 0.23	31.68 ± 0.40	23.15 ± 0.021	31.97 ± 0.39	28.52 ± 0.41	28.66 ± 0.24
cyclopropane	1.65 ± 0.01	1.17 ± 0.01	2.32 ± 0.02	1.52 ± 0.01	1.95 ± 0.02	2.63 ± 0.03	2.37 ± 0.02	2.21 ± 0.02
18:1	28.15 ± 1.91	23.80 ± 1.01	22.19 ± 1.93	22.40 ± 1.03	34.53 ± 0.89	23.61 ± 1.19	27.64 ± 0.18	27.04 ± 0.15
**LB 37 °C**								
12:0	0.18 ± 0.01	0.290 ±0.02	0.28 ± 0.01	0.28 ± 0.01	0.21 ± 0.02	0.28 ± 0.01	0.26 ± 0.02	0.25 ±0.01
14:0	3.55 ± 0.06	3.730 ± 0.04	3.72 ± 0.03	5.30 ± 0.07	1.88 ± 0.02	4.15 ± 0.03	2.84 ± 0.02	2.47 ± 0.03
16:0	41.53 ± 2.21	44.338 ± 1.90	41.67 ± 0.89	45.53 ± 0.15	40.94 ± 1.03	44.24 ± 1.14	43.67 ± 0.09	43.67 ± 0.08
18:0	0.68 ± 0.01	3.412 ± 0.06	0.65 ± 0.01	0.66 ± 0.02	1.51 ± 0.07	0.70 ± 0.02	0.80 ± 0.03	0.83 ± 0.02
16:1	32.20 ± 1.04	30.80 ± 0.09	34.14 ± 1.03	32.59 ± 0.08	27.57 ± 0.05	32.46 ± 0.08	30.06 ± 0.05	28.70 ± 0.06
cyclopropane	2.38 ± 0.02	2.49 ± 0.03	2.80 ± 0.01	2.78 ± 0.02	2.19 ± 0.07	2.43 ± 0.06	2.19 ± 0.04	2.83 ± 0.01
18:1	19.48 ± 0.06	14.94 ± 0.05	16.45 ± 0.09	12.42 ± 0.04	25.72 ± 0.09	15.38 ± 0.08	20.18 ± 1.01	21.25 ± 0.09

**Table 4:** Multicopy suppressors of Δ(*lapD clsA*) bacteria identify genes whose products can repress fatty acid biosynthesis or titrate elevated PG levels.

Gene	Function
*accC*	biotin carboxylase subunit of the ACC enzyme
*glnB*	the PII-1 protein-regulator of nitrogen metabolism and fatty acid via its interaction with the biotin carboxylase/biotin carboxyl carrier protein
*psd*	phosphatidylserine decarboxylase required for the formation of phosphatidylethanolamine
*tesD*	putative thioesterase with a hot-dog fold
*pspC*	lipoprotein, positive regulator of the *pspABCDE* operon, substrate of FtsH
*metQ*	lipoprotein, required for methionine uptake
*mliC*	lipoprotein, multicopy suppressor of Δ*lapB*, inhibitor of c-type lysozyme

**Table 5:** Bacterial strains and plasmids used in this study.

**Strains**	**Genotype**	**Reference**
W3110	λ^−^, *IN* (*rrnD-rrnE*)*1, rph-1*	CGSC, Yale
GK1111	W3110 Δ*lac*	[17]
SR7106	GK1111 *clsA*<>*aph*	This study
SR25204	GK1111 *lapD*<>*frt*	[14]
SR25209	SR25204 *lapD*<>*frt clsA*<>*aph*	This study
SR25533	SR25204 *lapD*<>*frt clsA*<>*aph pgsA* I99N	This study
SR25534	SR25204 *lapD*<>*frt clsA*<>*aph pgsA* T7A	This study
SR25537	SR25204 *lapD*<>*frt clsA*<>*aph pgsA* T60A	This study
SR25535	SR25204 *lapD*<>*frt clsA*<>*aph cdsA* D249G	This study
SR25532	SR25204 *lapD*<>*frt clsA*<>*aph murD* M407V	This study
SR25538	SR25204 *lapD*<>*frt clsA*<>*aph pgsA* T60P	This study
RU857	Δ*pgsA* Δ(*clsABC*) *lpp*2	[69]
SR25228	SR25209 *lapD*<>*frt clsA*<>*aph* + p*accC*^+^	This study
SR25229	SR25209 *lapD*<>*frt clsA*<>*aph* + p*psd*^+^	This study
SR25230	SR25209 *lapD*<>*frt clsA*<>*aph* + p*metQ*^+^	This study
SR25231	SR25209 *lapD*<>*frt clsA*<>*aph* + p*glnB^+^*	This study
SR25232	SR25209 *lapD*<>*frt clsA*<>*aph* + p*pspC^+^*	This study
SR25233	SR25209 *lapD*<>*frt clsA*<>*aph* + p*tesD^+^*	This study
SR25540	W3110 *pgsA* T60A Tn*10*	This study
SR25548	W3110 *cdsA* D249A Tn*10*	This study
SR25874	W3110 + pCA24N	[14]
SR23799	SR25204 *lapD*<>*frt* + pCA24N	[14]
SR10488	MG1655 Muc ts62 Mud 5005	[70]
SR23769	SR10488 *lapD*<>*frt clsA*<>*aph*	This study
**Plasmids**	**Genotype**	**Reference**
pCA24N	IPTG-inducible expression vector cm^R^	[41]
pKD13	*oriR6K_g_*, *bla*(Amp^R^), *kan*, *rgnB*(Ter)	[71]
pKD46	*araBp*-*gam*-*bet*-*exo*, *bla*(Amp^R^), *repA101*(ts)	[71]
pCP20	ts replicon with inducible FLP recombinase	[72]
pMBL18	p15A replicon amp^R^	[73]
pSR23502	*tesD*^+^ in pMBL18 amp^R^	This study
pSR23505	*tesD*^+^ in pCA24N cm^R^	This study
JW3224	*accC*^+^ in pCA24N cm^R^	[41]
JW0193	*metQ*^+^ in pCA24N cm^R^	[41]
JW2537	*glnB*^+^ in pCA24N cm^R^	[41]
JW1299	*pspC*^+^ in pCA24N cm^R^	[41]
JW4121	*psd*^+^ in pCA24N cm^R^	[41]
pREG	cosmid cloning vector	[74]
pSR25577	*pgsA*^+^ in pREG amp^R^	This study
pSR25590	*cdsA*^+^ in pREG amp^R^	This study
pSR25596	*murD*^+^ in pREG amp^R^	This study

## Data Availability

The original contributions presented in this study are included in the article. Further inquiries can be directed to the corresponding authors.

## References

[1] Nikaido H. (2003). Molecular basis of bacterial outer membrane permeability revisited. Microbiol. Mol. Biol. Rev..

[2] Klein G., Wieczorek A., Szuster M., Raina S. (2022). Checkpoints that regulate balanced biosynthesis of lipopolysaccharide and its essentiality in *Escherichia coli*. Int. J. Mol. Sci..

[3] Zhang Y.M., Rock C.O. (2008). Membrane lipid homeostasis in bacteria. Nat. Rev. Microbiol..

[4] Ogura T., Inoue K., Tatsuta T., Suzaki T., Karata K., Young K., Su L.H., Fierke C.A., Jackman J.E., Raetz C.R.H. (1999). Balanced biosynthesis of major membrane components through regulated degradation of the committed enzyme of lipid A biosynthesis by the AAA protease FtsH (HflB) in *Escherichia coli*. Mol. Microbiol..

[5] Klein G., Kobylak N., Lindner B., Stupak A., Raina S. (2014). Assembly of lipopolysaccharide in *Escherichia coli* requires the essential LapB heat shock protein. J. Biol. Chem..

[6] Mohan S., Kelly T.M., Eveland S.S., Raetz C.R., Anderson M.S. (1994). An *Escherichia coli* gene (*fabZ*) encoding (3*R*)-hydroxymyristoyl acyl carrier protein dehydrase. Relation to *fabA* and suppression of mutations in lipid A biosynthesis. J. Biol. Chem..

[7] Sorensen P.G., Lutkenhaus J., Young K., Eveland S.S., Anderson M.S., Raetz C.R.H. (1996). Regulation of UDP-3-*O*-[*R*-3-hydroxymyristoyl]-*N*-acetylglucosamine deacetylase in *Escherichia coli*. The second enzymatic step of lipid A biosynthesis. J. Biol. Chem..

[8] Biernacka D., Gorzelak P., Klein G., Raina S. (2020). Regulation of the first committed step in lipopolysaccharide biosynthesis catalyzed by LpxC requires the essential protein LapC (YejM) and HslVU protease. Int. J. Mol. Sci..

[9] Shu S., Mi W. (2022). Regulatory mechanisms of lipopolysaccharide synthesis in *Escherichia coli*. Nat. Commun..

[10] Raetz C.R.H., Reynolds C.M., Trent M.S., Bishop R.E. (2007). Lipid A modification systems in Gram-negative bacteria. Annu. Rev. Biochem..

[11] Klein G., Raina S. (2017). Small regulatory bacterial RNAs regulating the envelope stress response. Biochem. Soc. Trans..

[12] Valvano M.A. (2022). Remodelling of the Gram-negative bacterial Kdo_2_-lipid A and its functional implications. Microbiology.

[13] Wieczorek A., Sendobra A., Maniyeri A., Sugalska M., Klein G., Raina S. (2022). A new factor LapD is required for the regulation of LpxC amounts and lipopolysaccharide trafficking. Int. J. Mol. Sci..

[14] Jeschke M., Ayyolath A., Maniyeri A., Raina S., Klein G. (2025). Coordinated biosynthesis of essential cell envelope components: Lipopolysaccharide and fatty acids requires LapD, acyl carrier protein, and fully hexaacylated lipid A. Int. J. Mol. Sci..

[15] Raetz C.R.H., Whitfield C. (2002). Lipopolysaccharide endotoxins. Annu. Rev. Biochem..

[16] Doerrler W.T., Gibbons H.S., Raetz C.R. (2004). MsbA-dependent translocation of lipids across the inner membrane of *Escherichia coli*. J. Biol. Chem..

[17] Klein G., Lindner B., Brabetz W., Brade H., Raina S. (2009). *Escherichia coli* K-12 suppressor-free mutants lacking early glycosyltransferases and late acyltransferases: Minimal lipopolysaccharide structure and induction of envelope stress response. J. Biol. Chem..

[18] Douglass M.V., Cléon F., Trent M.S. (2021). Cardiolipin aids in lipopolysaccharide transport to the Gram-negative outer membrane. Proc. Natl. Acad. Sci. USA.

[19] Gorzelak P., Klein G., Raina S. (2021). Molecular basis of essentiality of early critical steps in the lipopolysaccharide biogenesis in *Escherichia coli* K-12: Requirement of MsbA, cardiolipin, LpxL, LpxM and GcvB. Int. J. Mol. Sci..

[20] Guchhait R.B., Polakis S.E., Dimroth P., Stoll E., Moss J., Lane M.D. (1974). Acetyl coenzyme A carboxylase system of *Escherichia coli*. Purification and properties of the biotin carboxylase, carboxyltransferase, and carboxyl carrier protein components. J. Biol. Chem..

[21] Abdel-Hamid A.M., Cronan J.E. (2007). Coordinate expression of the acetyl coenzyme A carboxylase genes, *accB* and *accC*, is necessary for normal regulation of biotin synthesis in *Escherichia coli*. J. Bacteriol..

[22] Parsons J.B., Rock C.O. (2013). Bacterial lipids: Metabolism and membrane homeostasis. Prog. Lipid Res..

[23] Yao J., Rock C.O. (2013). Phosphatidic acid synthesis in bacteria. Biochim. Biophys. Acta.

[24] Coleman J. (1992). Characterization of the *Escherichia coli* gene for 1-acyl-*sn*-glycerol-3-phosphate acyltransferase (*plsC*). Mol. Gen. Genet..

[25] Dowhan W. (1997). CDP-diacylglycerol synthase of microorganisms. Biochim. Biophys. Acta..

[26] Romantsov T., Guan Z., Wood J.M. (2009). Cardiolipin and the osmotic stress responses of bacteria. Biochim. Biophys. Acta.

[27] Renner L.D., Weibel D.B. (2012). MinD and MinE interact with anionic phospholipids and regulate division plane formation in *Escherichia coli*. J. Biol. Chem..

[28] Corey R.A., Pyle E., Allen W.J., Watkins D.W., Casiraghi M., Miroux B., Arechaga I., Politis A., Collinson I. (2018). Specific cardiolipin-SecY interactions are required for proton-motive force stimulation of protein secretion. Proc. Natl. Acad. Sci. USA.

[29] Icho T., Sparrow C.P., Raetz C.R. (1985). Molecular cloning and sequencing of the gene for CDP-diglyceride synthetase of *Escherichia coli*. J. Biol. Chem..

[30] Pluschke G., Hirota Y., Overath P. (1978). Function of phospholipids in *Escherichia coli*. Characterization of a mutant deficient in cardiolipin synthesis. J. Biol. Chem..

[31] Tropp B.E. (1997). Cardiolipin synthase from *Escherichia coli*. Biochim. Biophys. Acta.

[32] Zhu K., Zhang Y.M., Rock C.O. (2009). Transcriptional regulation of membrane lipid homeostasis in *Escherichia coli*. J. Biol. Chem..

[33] Usui M., Sembongi H., Marsuzaki H., Matsumoto K., Shibuya I. (1994). Primary structures of the wild-type and mutant alleles encoding the phosphatidylglycerophosphate synthase of *Escherichia coli*. J. Bacteriol..

[34] Suzuki E., Mizushima T., Sekimizu K. (1997). Alteration of fatty acid composition in a *pgsA3* mutant of *Escherichia coli*. Biol. Pharm. Bull..

[35] Akimitsu N., Mizushima T., Suzuki E., Miki T., Sekimizu K. (1996). Growth phenotypes of *Escherichia coli* carrying a mutation of acidic phospholipid synthesis. Biol. Pharm. Bull..

[36] Miyazaki C., Kuroda M., Ohta A., Shibuya I. (1985). Genetic manipulation of membrane phospholipid composition in *Escherichia coli*: *pgsA* mutants defective in phosphatidylglycerol synthesis. Proc. Natl. Acad. Sci. USA.

[37] Sparrow C.P., Raetz C.R. (1985). Purification and properties of the membrane-bound CDP-diglyceride synthetase from *Escherichia coli*. J. Biol. Chem..

[38] Bouhss A., Dementin S., Parquet C., Mengin-Lecreulx D., Bertrand J.A., Le Beller D., Dideberg O., van Heijenoort J., Blanot D. (1999). Role of the ortholog and paralog amino acid invariants in the active site of the UDP-MurNAc-L-alanine:D-glutamate ligase (MurD). Biochemistry.

[39] Shibuya I., Miyazaki C., Ohta A. (1985). Alteration of phospholipid composition by combined defects in phosphatidylserine and cardiolipin synthases and physiological consequences in *Escherichia coli*. J. Bacteriol..

[40] Vadia S., Tse J.L., Lucena R., Yang Z., Kellogg D.R., Wang J.D., Levin P.A. (2017). Fatty acid availability sets cell envelope capacity and dictates microbial cell size. Curr. Biol..

[41] Kitagawa M., Ara T., Arifuzzaman M., Ioka-Nakamichi T., Inamoto E., Toyonaga H., Mori H. (2005). Complete set of ORF clones of *Escherichia coli* ASKA library (a complete set of *E. coli* K-12 ORF archive): Unique resources for biological research. DNA Res..

[42] Roncero C., Casadaban M.J. (1992). Genetic analysis of the genes involved in synthesis of the lipopolysaccharide core in *Escherichia coli* K-12: Three operons in the *rfa* locus. J. Bacteriol..

[43] Vasudevan S.G., Gedye C., Dixon N.E., Cheah E., Carr P.D., Suffolk P.M., Jeffrey P.D., Ollis D.L. (1994). *Escherichia coli* PII protein: Purification, crystallization and oligomeric structure. FEBS Lett..

[44] Carr P.D., Cheah E., Suffolk P.M., Vasudevan S.G., Dixon N.E., Ollis D.L. (1996). X-ray structure of the signal transduction protein from *Escherichia coli* at 1.9 Å. Acta Crystallogr. D Biol. Crystallogr..

[45] Rodionova I.A., Goodacre N., Babu M., Emili A., Uetz P., Saier M.H. (2018). The nitrogen regulatory PII protein (GlnB) and *N*-acetylglucosamine 6-phosphate epimerase (NanE) allosterically activate alucosamine 6-phosphate deaminase (NagB) in *Escherichia coli*. J. Bacteriol..

[46] Gerhardt E.C., Rodrigues T.E., Müller-Santos M., Pedrosa F.O., Souza E.M., Forchhammer K., Huergo L.F. (2015). The bacterial signal transduction protein GlnB regulates the committed step in fatty acid biosynthesis by acting as a dissociable regulatory subunit of acetyl-CoA carboxylase. Mol. Microbiol..

[47] Singh S., Darwin A.J. (2011). FtsH-dependent degradation of phage shock protein C in *Yersinia enterocolitica* and *Escherichia coli*. J. Bacteriol..

[48] Tyhach R.J., Hawrot E., Satre M., Kennedy E.P. (1979). Increased synthesis of phosphatidylserine decarboxylase in a strain of *Escherichia coli* bearing a hybrid plasmid. Altered association of enzyme with the membrane. J. Biol. Chem..

[49] Goodall E.C.A., Isom G.L., Rooke J.L., Pullela K., Icke C., Yang Z., Boelter G., Jones A., Warner I., Da Costa R. (2021). Loss of YhcB results in dysregulation of coordinated peptidoglycan, LPS and phospholipid synthesis during *Escherichia coli* cell growth. PLoS Genet..

[50] Lu Y.H., Guan Z., Zhao J., Raetz C.R. (2011). Three phosphatidylglycerol-phosphate phosphatases in the inner membrane of *Escherichia coli*. J. Biol. Chem..

[51] Matsumoto K. (2001). Dispensable nature of phosphatidylglycerol in *Escherichia coli*: Dual roles of anionic phospholipids. Mol. Microbiol..

[52] Kurita K., Kato F., Shiomi D. (2020). Alteration of membrane fluidity or phospholipid composition perturbs rotation of MreB complexes in *Escherichia coli*. Front. Mol. Biosci..

[53] Renner L.D., Weibel D.B. (2011). Cardiolipin microdomains localize to negatively curved regions of *Escherichia coli* membranes. Proc. Natl. Acad. Sci. USA.

[54] Li G., Hamamoto K., Kitakawa M. (2012). Inner membrane protein YhcB interacts with RodZ involved in cell shape maintenance in *Escherichia coli*. ISRN Mol. Biol..

[55] Weart R.B., Lee A.H., Chien A.C., Haeusser D.P., Hill N.S., Levin P.A. (2007). A metabolic sensor governing cell size in bacteria. Cell.

[56] Yao Z., Davis R.M., Kishony R., Kahne D., Ruiz N. (2012). Regulation of cell size in response to nutrient availability by fatty acid biosynthesis in *Escherichia coli*. Proc. Natl. Acad. Sci. USA.

[57] Sekimizu K., Kornberg A. (1988). Cardiolipin activation of DnaA protein, the initiation protein of replication in *Escherichia coli*. J. Biol. Chem..

[58] Newman G., Crooke E. (2000). DnaA, the initiator of *Escherichia coli* chromosomal replication, is located at the cell membrane. J. Bacteriol..

[59] Furse S., Wienk H., Boelens R., de Kroon A.I., Killian J.A. (2015). *E. coli* MG1655 modulates its phospholipid composition through the cell cycle. FEBS Lett..

[60] James E.S., Cronan J.E. (2004). Expression of two *Escherichia coli* acetyl-CoA carboxylase subunits is autoregulated. J. Biol. Chem..

[61] Hauf W., Schmid K., Gerhardt E.C., Huergo L.F., Forchhammer K. (2016). Interaction of the nitrogen regulatory protein GlnB (P_II_) with biotin carboxyl carrier protein (BCCP) controls acetyl-CoA levels in the cyanobacterium *Synechocystis* sp. PCC 6803. Front. Microbiol..

[62] Brissette J.L., Weiner L., Ripmaster T.L., Model P. (1991). Characterization and sequence of the *Escherichia coli* stress-induced *psp* operon. J. Mol. Biol..

[63] Asai Y., Katayose Y., Hikita C., Ohta A., Shibuya I. (1989). Suppression of the lethal effect of acidic-phospholipid deficiency by defective formation of the major outer membrane lipoprotein in *Escherichia coli*. J. Bacteriol..

[64] Kikuchi S., Shibuya I., Matsumoto K. (2000). Viability of an *Escherichia coli pgsA* null mutant lacking detectable phosphatidylglycerol and cardiolipin. J. Bacteriol..

[65] Mehner D., Osadnik H., Lünsdorf H., Brüser T. (2012). The Tat system for membrane translocation of folded proteins recruits the membrane-stabilizing Psp machinery in *Escherichia coli*. J. Biol. Chem..

[66] Caswell B.T., de Carvalho C.C., Nguyen H., Roy M., Nguyen T., Cantu D.C. (2022). Thioesterase enzyme families: Functions, structures, and mechanisms. Protein Sci..

[67] Lennen R.M., Pfleger B.F. (2013). Modulating membrane composition alters free fatty acid tolerance in *Escherichia coli*. PLoS ONE.

[68] Voelker T.A., Davies H.M. (1994). Alteration of the specificity and regulation of fatty acid synthesis of *Escherichia coli* by expression of a plant medium-chain acyl-acyl carrier protein thioesterase. J. Bacteriol..

[69] Kawazura T., Matsumoto K., Kojima K., Kato F., Kanai T., Niki H., Shiomi D. (2017). Exclusion of assembled MreB by anionic phospholipids at cell poles confers cell polarity for bidirectional growth. Mol. Microbiol..

[70] Zakataeva N.P., Aleshin V.V., Tokmakova I.L., Troshin P.V., Livshits V.A. (1999). The novel transmembrane *Escherichia coli* proteins involved in the amino acid efflux. FEBS Lett..

[71] Datsenko K.A., Wanner B.L. (2000). One-step inactivation of chromosomal genes in *Escherichia coli* K-12 using PCR products. Proc. Natl. Acad. Sci. USA.

[72] Cherepanov P.P., Wackernagel W. (1995). Gene disruption in *Escherichia coli*: Tc^R^ and Km^R^ cassettes with the option of Flp-catalyzed excision of the antibiotic-resistance determinant. Gene.

[73] Nakano Y., Yoshida Y., Yamashita Y., Koga T. (1995). Construction of a series of pACYC-derived plasmid vectors. Gene.

[74] O’Connor M., Gesteland R.F., Atkins J.F. (1989). tRNA hopping: Enhancement by an expanded anticodon. EMBO J..

[75] Raina S., Missiakas D., Baird L., Kumar S., Georgopoulos C. (1993). Identification and transcriptional analysis of the *Escherichia coli htrE* operon which is homologous to *pap* and related pilin operons. J. Bacteriol..

[76] Dartigalongue C., Loferer H., Raina S. (2001). EcfE, a new essential inner membrane protease: Its role in the regulation of heat shock response in *Escherichia coli*. EMBO J..

[77] Kleckner N., Bender J., Gottesman S. (1991). Uses of transposons with emphasis on Tn*10*. Methods Enzymol..

[78] Wojtkiewicz P., Biernacka D., Gorzelak P., Stupak A., Klein G., Raina S. (2020). Multicopy suppressor analysis of strains lacking cytoplasmic peptidyl-prolyl *cis/trans* isomerases identifies three new PPIase activities in *Escherichia coli* that includes the DksA transcription factor. Int. J. Mol. Sci..

[79] Bligh E.G., Dyer W.J. (1959). A rapid method of total lipid extraction and purification. Can. J. Biochem. Physiol..

[80] Ames G.F. (1968). Lipids of *Salmonella typhimurium* and *Escherichia coli*: Structure and metabolism. J. Bacteriol..

[81] Politz M., Lennen R., Pfleger B. (2013). Quantification of bacterial fatty acids by extraction and methylation. Bio Protoc..

[82] Klein G., Wojtkiewicz P., Biernacka D., Stupak A., Gorzelak P., Raina S. (2020). Identification of substrates of cytoplasmic peptidyl-prolyl *cis/trans* isomerases and their collective essentiality in *Escherichia coli*. Int. J. Mol. Sci..

